# Cerebellar Modules and Their Role as Operational Cerebellar Processing Units

**DOI:** 10.1007/s12311-018-0952-3

**Published:** 2018-06-06

**Authors:** Richard Apps, Richard Hawkes, Sho Aoki, Fredrik Bengtsson, Amanda M. Brown, Gang Chen, Timothy J. Ebner, Philippe Isope, Henrik Jörntell, Elizabeth P. Lackey, Charlotte Lawrenson, Bridget Lumb, Martijn Schonewille, Roy V. Sillitoe, Ludovic Spaeth, Izumi Sugihara, Antoine Valera, Jan Voogd, Douglas R. Wylie, Tom J. H. Ruigrok

**Affiliations:** 1 School of Physiology, Pharmacology and Neuroscience, University of Bristol, Bristol, UK; 2 Hotchkiss Brain Institute, University of Calgary, Calgary, Canada; 3 Neurobiology Research Unit, Okinawa Institute of Science and Technology, Onna, Japan; 4 Department of Neuroscience, Erasmus MC Rotterdam, Rotterdam, the Netherlands; 5 Department of Experimental Medical Sciences, Lund University, Lund, Sweden; 6 Department of Pathology & Immunology, Baylor College of Medicine, Houston, TX USA; 7 Department of Neuroscience, Baylor College of Medicine, Houston, TX USA; 8 Jan and Dan Duncan Neurological Research Institute at Texas Children’s Hospital, Houston, TX USA; 9 Department of Neuroscience, University of Minnesota, Minneapolis, MN USA; 10 Institut des Neurosciences Cellulaires et Intégratives, CNRS, Université de Strasbourg, Strasbourg, France; 11 Program in Developmental Biology, Baylor College of Medicine, Houston, TX USA; 12 Department of Systems Neurophysiology, Graduate School of Medical and Dental Sciences, Tokyo Medical and Dental University, Tokyo, Japan; 13 Neuroscience and Mental Health Institute, University of Alberta, Edmonton, AB Canada

**Keywords:** Cerebellum, Purkinje cells, Compartments, Climbing fibers, Mossy fibers, Zebrin, Aldolase C, Functional organization, Longitudinal stripes, Microzones

## Abstract

The compartmentalization of the cerebellum into modules is often used to discuss its function. What, exactly, can be considered a module, how do they operate, can they be subdivided and do they act individually or in concert are only some of the key questions discussed in this consensus paper. Experts studying cerebellar compartmentalization give their insights on the structure and function of cerebellar modules, with the aim of providing an up-to-date review of the extensive literature on this subject. Starting with an historical perspective indicating that the basis of the modular organization is formed by matching olivocorticonuclear connectivity, this is followed by consideration of anatomical and chemical modular boundaries, revealing a relation between anatomical, chemical, and physiological borders. In addition, the question is asked what the smallest operational unit of the cerebellum might be. Furthermore, it has become clear that chemical diversity of Purkinje cells also results in diversity of information processing between cerebellar modules. An additional important consideration is the relation between modular compartmentalization and the organization of the mossy fiber system, resulting in the concept of modular plasticity. Finally, examination of cerebellar output patterns suggesting cooperation between modules and recent work on modular aspects of emotional behavior are discussed. Despite the general consensus that the cerebellum has a modular organization, many questions remain. The authors hope that this joint review will inspire future cerebellar research so that we are better able to understand how this brain structure makes its vital contribution to behavior in its most general form.

## Introduction

It is difficult to give a consensus of informed opinion because, although there is much informed opinion, there is rather little consensus. David Colquhoun (1971) Lectures on Biostatistics. Oxford, UK: Clarendon Press.

**Fig. 1:**
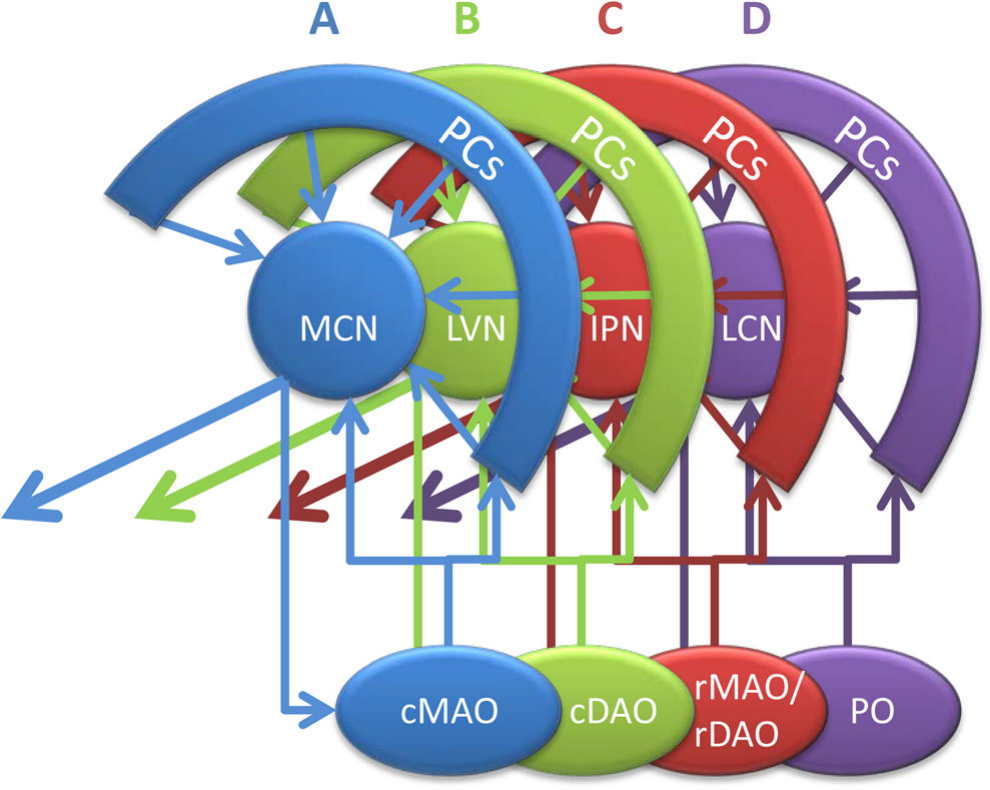
Simplified diagram illustrating the four main modules of the right cerebellum seen from medial. The elementary modular connections are based on the projection of longitudinally arranged strips of Purkinje cells (PCs) to four main target nuclei and their olivocerebellar input from selective inferior olivary subnuclei. As such two vermal Purkinje cell zones (A and B) are recognized, together with their respective targets, the medial cerebellar nucleus (MCN) and lateral vestibular nucleus (LVN) and their sources of climbing fibers, caudal parts of the medial accessory (cMAO) and dorsal accessory (cDAO) olives, respectively. The C zones of the paravermis targets the interposed nuclei (IPN) and receives climbing fibers from the rostral (r) MAO and rDAO, while the D zones targets the lateral cerebellar nucleus (LCN) and receive from the principal olive (PO). Note that olivary subnuclei are also reciprocally connected according the same scheme. The interconnected olivocorticonuclear entity is referred to as module and each have a specific output. All modules (apart from the B module) have been further subdivided. Note that the modules of the vestibulocerebellum are not indicated in this diagram. Modified after Ruigrok [6]

The cerebellum has long been considered as a uniform structure with well-organized in- and output relations that ultimately serves a particular adaptive control function that is mainly, if not completely, used for coordinating, modifying, adapting, and learning motor functions [1, 2]. By now, we have learned that the idea of an operational uniform cerebellar cortex needs to be revised [3]. In addition, the functional extent of cerebellar influence extends to cognitive, affective, and autonomic domains [4, 5]. Yet, although not completely resolved, one consideration that is generally accepted is that the basic operational unit is the cerebellar module. Each cerebellar module includes a longitudinal, i.e., (para-)sagittally organized, zone of Purkinje cells (PCs) in the cerebellar cortex that receives common climbing fiber input from a particular region of the inferior olive, and in turn, the same PCs target a discrete part of the cerebellar nuclei. This part of the nuclei is also targeted by collaterals of the same olivocerebellar axons that provide the climbing fibers to the zone of PCs, and harbors a population of small GABAergic neurons that project back to the same part of the inferior olive. This precise olivo-cortico-nuclear circuitry forms the core of individual cerebellar modules (Fig. 1). The basic cerebellar modules, A, B, C, and D, as defined by Voogd [7] have now each been subdivided into several smaller entities and in some cases, based on similar peripheral receptive fields, these have been shown to comprise yet smaller units, termed microzones, which are the cortical component of micromodules [6, 8–12].

Several decades ago, it became clear that the apparent uniformity of the cerebellar cortex masked underlying differences in the expression of a multitude of genetic markers in a broad transverse and finer parasagittally organized patterns, which are commonly referred to as stripes [8]. Much work has been devoted to describe the organization of the anatomically defined zones in relation to these biochemically defined stripes [13]. This interest has gained new impetus given the additional finding that differences in physiological properties can be related to this biochemical heterogeneity [14–17]. Such a finding raises the important possibility that individual cerebellar modules may not be uniform in their operation [3]. The current paper brings together up-to-date views on cerebellar modules. The general approach is at a systems level in order to understand the neural circuit basis of cerebellar modules and to establish to what extent they are functional entities and can fulfill functions that are independent of other modules.

Jan Voogd, who first used the term “cerebellar module” to describe the basic operational unit of the cerebellum, provides an historical synopsis. Izumi Sugihara subsequently reviews his work on the relation between modules and several biochemical markers. His detailed scheme of the relation of olivocerebellar organization and the aldolase C (zebrin II) pattern is now widely used, but he also points to the shortcomings of the aldolase C pattern and the great potential that additional markers may have in studying both the development and the adult organization of cerebellar modules. Doug Wylie uses the vestibulocerebellum system in the pigeon to examine sagittally organized zones of PCs and how they modulate their activity in response to optic flow. Although these zones are present in lobule IXcd and in lobule X, their relation to the zebrin pattern of stripes differs, as there is no distinctive pattern in lobule X, whereas the same functional zones cover adjacent stripes of zebrin II-positive (ZII+) and zebrin II-negative (ZII-) PCs in lobule IX. This raises the important issue that zebrin alone is insufficient as a marker to describe the functional heterogeneity of PCs. Richard Hawkes subsequently explores the extent to which cerebellar modules can be divisible into their smallest processing units, leading to the idea of the “cerebellar quantum.” As such, the cerebellar cortex may be made up of short strips or microzones (i.e., positioned within an anatomically defined zone or biochemically defined stripe) or, maybe, elongated patches, which, together, may comprise several thousands of individual processing units. Parallel processing power, positional coding, improving signal-to-noise ratios, and functional processing diversity are potential advantages of such modular processing. The question of what constitutes the basic functional unit of the cerebellum is also asked by Fredrik Bengtsson and Henrik Jörntell. However, they address this important question from a systems level physiological perspective and propose that the fundamental unit of the cerebellar cortex is a population of PCs located within a given microzone, working together as a “super PC.” In pinpointing the cerebellar quantum (Hawkes) or the super PC (Bengtsson and Jörntell), both sections touch upon the role of mossy fiber afferents that show a more prominent transverse orientation but also adhere to modular organizational principles. This aspect is further discussed by Roy Sillitoe and colleagues who explore the relation between the organization of the mossy fiber systems, granule cells, and cortical interneurons.

These initial sections mostly deal with the anatomical foundations of the cerebellar modular functionality and are followed by sections that concentrate on their physiological properties. Martijn Schonewille reviews differences in several physiological properties of PCs with different molecular signatures. This significant recent development in cerebellar physiology is also highlighted by Gang Chen and Tim Ebner, who further explore the physiological and functional differences of modules based on ZII+ and ZII− stripes. Philippe Isope, Ludovic Spaeth, and Antoine Valera, on the other hand, return to the effect of mossy fiber input on plasticity within modular circuits and propose that modular identity may not be rigid but adaptable.

Exploring the fate of cerebellar modular output, Sho Aoki and Tom Ruigrok survey how this output is distributed and used by other areas—does the output from individual modules remain separated or can the outputs of different modules converge to be jointly processed in common receiving areas? Finally, Richard Apps and colleagues review recent developments on cerebellar involvement in emotional behavior. In line with the ideas developed in the previous section, they call attention to a body of evidence that the various modular constituents of the vermal A zone are connected to widespread brainstem and diencephalic (limbic) areas. They suggest that different components of the A module (possibly relating to micromodules) may carry out different, but orchestrated, aspects of an integrated emotional response.

## Defining Cerebellar Modules (J. Voogd)

**Fig. 2:**
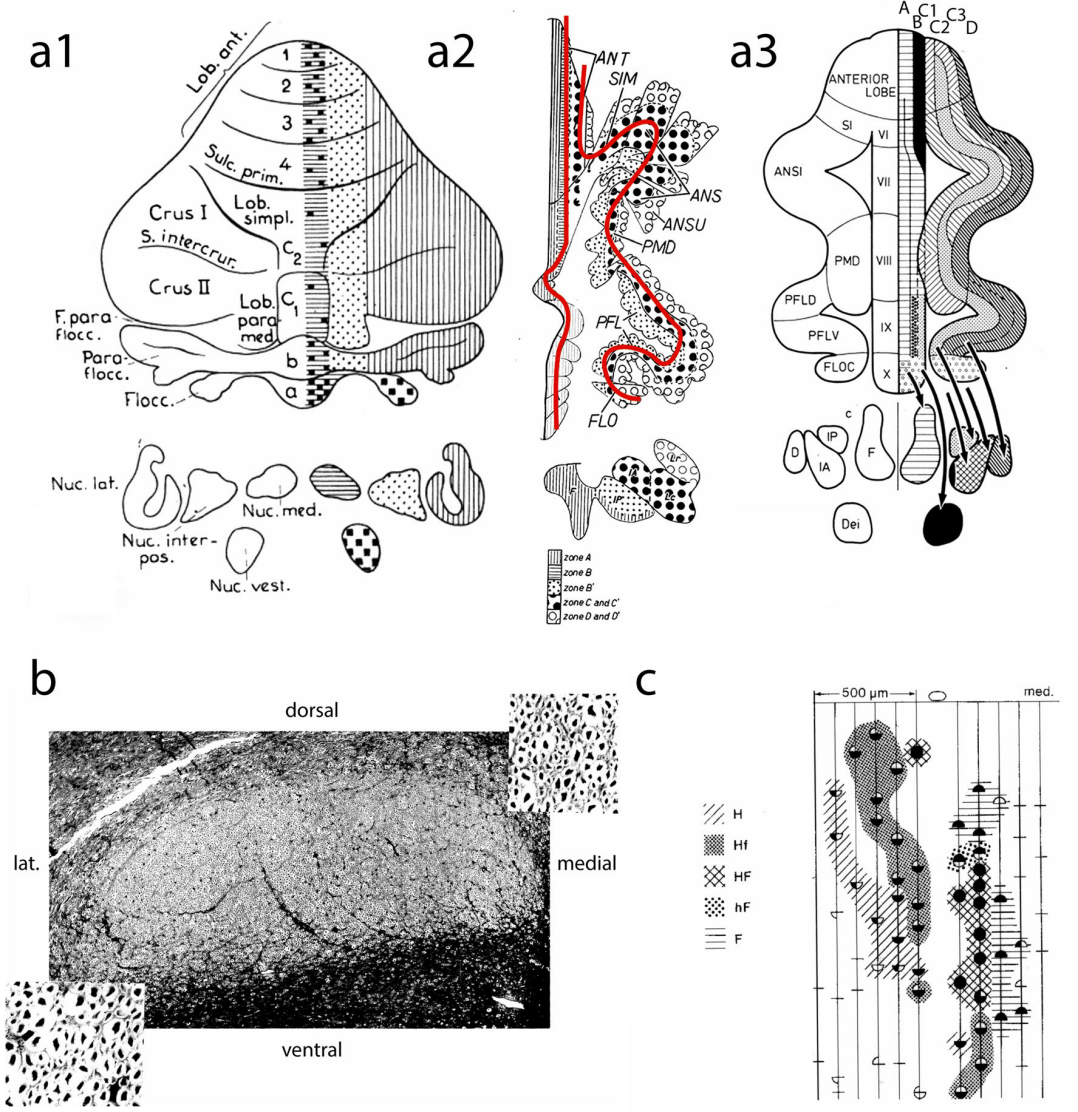
**a1** Diagram of the corticonuclear projection of the cerebellum, showing the vermal, intermediate, and lateral zones of Jansen and Brodal [24]. Nomenclature of the lobules according to Bolk [25]. **a2** Diagram of the flattened cerebellar cortex of the cat showing the corticonuclear projection (after Voogd [26]). The red lines indicate the direction of the folial chains of vermis and hemisphere. **a3** Corticonuclear projection shown in diagrams of the flattened cerebellar cortex of the cat from Groenewegen et al. [19]. **b** Superior cerebellar peduncle of the cat, Häggqvist stain. Note small myelinated fibers in the medial third and coarse fibers in lateral two-thirds [after 24]. **c** Microzones with different climbing fiber inputs in the B zone of the cerebellum of the cat. Stimulation of the ipsilateral and contralateral ulnar and sciatic nerves results in Purkinje cells with similar responses in microzones as indicated by different hatching and stippling: H (hindlimb), Hf (mainly hindlimb), HF (hind- and forelimb), hF (mainly forelimb), F (forelimb), after Andersson and Oscarsson [27]. *ANS*, *ANSI* ansiform lobule; *ANSU* ansula; *D* dentate nucleus; *Dei* Deiters nucleus; *F* fastigial nucleus; *F*. *parafloc* parafloccular fissure; *FLO*, *FLOC* flocculus; *IA* anterior interposed nucleus; *IP* posterior interposed nucleus; *Lc*. *Lateral nucleus* pars convexa; *Lob*. *Paramed* paramedian lobule; *Lob*.*ant*, *ANT* anterior lobe; *Lob*.*simpl* simple lobule; *Lr*, *lateral nucleus* pars rotunda; *Nuc*.*interpos* interposed nucleus; *Nuc*.*lat* lateral nucleus; *Nuc*.*med*. medial nucleus; *Nuc*.*vest*. vestibular nucleus; *Parafloc* paraflocculus; *PFL*(D,V) paraflocculus (dorsalis, ventralis); *PMD* paramedian lobule; *S*.*intercrur* intercrural sulcus; *SIM*, *SI* primary fissure simplex lobul; *Sulc*.*prim*

The term “modules” was first used for Purkinje cell zones defined by their cerebellar and vestibular target nuclei and their climbing fiber afferents by Voogd and Bigaré [18] in a paper read at a meeting in Montreal. Our paper was based on the work of Groenewegen et al. [19] and Bigaré [20]. Cerebellar modules, however, were recognized before this term was used by us. In Brodal’s [21] study of the olivocerebellar projection in the cat and Jansen and Brodal’s [22, 23] studies of the corticonuclear projection, the lobules were the units or modules in their description. As a byproduct, they described an intermediate zone, located in the anterior lobe hemisphere, lateral to the vermis, that, like the vermis, received an olivocerebellar projection from the accessory olives but projected to the interposed nucleus. This was the first definition of a longitudinal Purkinje cell zone as we know it today. Attempts to extrapolate the intermediate zone to more posterior parts of the cerebellum failed, because the authors did not recognize the loops in the folial chains in the posterior cerebellum (Fig. 2(a1)).

My contribution to the distinction of longitudinal Purkinje cell zones was based on the following considerations [26, 28]. Bolk’s [25] description of the cerebellar vermis and hemisphere as folial chains with ansiform and (para-) floccular loops defined the topography of the Purkinje cell zones (Fig. 2(a2)). The distinction of anterior and posterior subdivisions in Brunner’s [29] interposed nucleus and of dorsal and ventral subdivisions of the lateral cerebellar nucleus as target nuclei of the zones was based on the localization of the relatively small myelinated fibers from the posterior interposed nucleus in the medial one-third and of the larger fibers from the anterior interposed and the dorsal part of the lateral cerebellar nucleus in the lateral two-thirds of the brachium conjunctivum [30] (Fig. 2**(**b)). Finally, the observation of compartments in the white matter that channeled the Purkinje cell axons to their target nuclei provided an intrinsic coordinate system for the zones. The innervation of Purkinje cell zones by specific subdivisions of the inferior olive followed from the localization of their olivocerebellar fibers in the corresponding white matter compartments [7]. Their termination as longitudinal zones of climbing fibers was first shown by Courville et al. [31], the organizer of the Montreal meeting. As a consequence, seven zones were distinguished (Fig. 2(a3)). Two were located in the vermis. The medial A zone projecting to the fastigial nucleus, the lateral B zone to Deiters’ nucleus. In the hemisphere, the C1 and C3 zones that connect with the anterior interposed nucleus and C2 that projects to the posterior interposed nucleus replaced Brodal and Jansen’s intermediate zone. The hemisphere was found to be composed of the two D zones that project to different parts of the dentate nucleus.

This simple zonal pattern was found to be inadequate after Hawkes and Leclerc’s [32] discovery of the “stripy”’ distribution of ZII+ and ZII− PCs. Apart from the identification of the B, C1, and C3 Purkinje cell zones as being positioned within ZII− stripes and the C2, D1, and D2 zones within ZII+ stripes, a number of narrow, ZII+ “satellite bands” were found to be present. These narrow bands, like their broad counterparts, are characterized by their climbing fiber afferents and, presumably, also by their corticonuclear projection [10, 33, 34]. The reconstruction of this more complicated map now serves as the standard reference for the description of zonal organization of the cerebellum [13].

Where the history of the Purkinje cell zones goes back to the early twentieth century [35], microzones made their appearance much later. They were first identified in the B zone of the cerebellum of the cat by Andersson and Oscarsson [27]. They consist of 50-mm-long and at least 200-μm-wide strips of PCs sharing the same climbing fiber receptive fields. The five microzones distinguished in the B zone differ in their input from forelimb or hindlimb nerves or a mixture of these nerves and the short or long latency of the response (Fig. 2(c)). The somatotopical localization in the B zone with the forelimb medially and the hindlimb laterally earlier was described by Oscarsson and Uddenberg [36]. Evoked potentials from the dorsal spino-olivary climbing fiber system [37] and the exteroceptive component of the cuneocerebellar mossy fiber system [38] are distributed in a similar, but more detailed microzonal pattern in the anterior lobe C3 zone of the cerebellum of the cat [39]. Overall, mossy fibers innervating these microzones had receptive fields resembling the climbing fiber receptive field defining that microzone [40].

What is the morphological basis for the microzones? The termination of mossy fibers in narrow longitudinal aggregates of rosettes in the granular layer was already described by Scheibel [41]. A similar, microzone-like distribution of individual climbing fibers was reported by Sugihara et al. [42]. The significance of the termination of mossy fibers in multiple longitudinal strips of mossy fiber terminals is difficult to understand, because this pattern would be erased by the parallel fibers [43]. Microzones, defined by their cutaneous receptive field of olivary mediated complex spike responses, thus far, only have been identified in the C1 and C3 zones of the anterior lobe. The microzone-like terminations of single or small groups of climbing and mossy fibers are present in the entire cerebellum. It would be interesting to know what these thousands or even millions of microzones in other parts of the cerebellum represent.

## Molecular Labeling of Cerebellar Topographic Modules (I. Sugihara)

### Correlation Between Molecular Expression and the Cerebellar Modular Structure

Cerebellar modules are basically defined by topographic axonal connections between subareas of the three major structures of the cerebellar system: cerebellar cortex, cerebellar nuclei, and inferior olive [6, 18]. Thus, the cerebellar system is compartmentalized into multiple modules, which are supposed to be the bases of different functional localization. These compartments, particularly those in the cerebellar cortex, are often characterized by the presence of a different profile of molecular expression, which can conversely be used to label compartments specifically.

Heterogeneous expression of some molecules, cell adhesion molecules in particular, has a significant role in the control of the aggregation and rearrangement of Purkinje cell subsets, and target specification and synaptic formation of afferent and efferent axons, which are essential for cerebellar module formation. However, the functional significance of the heterogeneous expression of many other molecules has not been clarified yet. The heterogeneous expression of molecules in cerebellar modules persists until adulthood in some cases, or newly emerges during the postnatal developmental stages and stays until adulthood in other cases. The correlation between the molecular expression pattern and the functional cerebellar modular organization is highly variable among molecules but usually conserved among individual animals for each molecule. Therefore, molecular expression pattern can be a useful genetic and histological tool to examine the anatomy and physiology of cerebellar modules. Its positional correlation to the cerebellar modular organization has been clarified for several molecules.

### Zebrin (Aldolase C) Expression in Cerebellar Modules

**Fig. 3:**
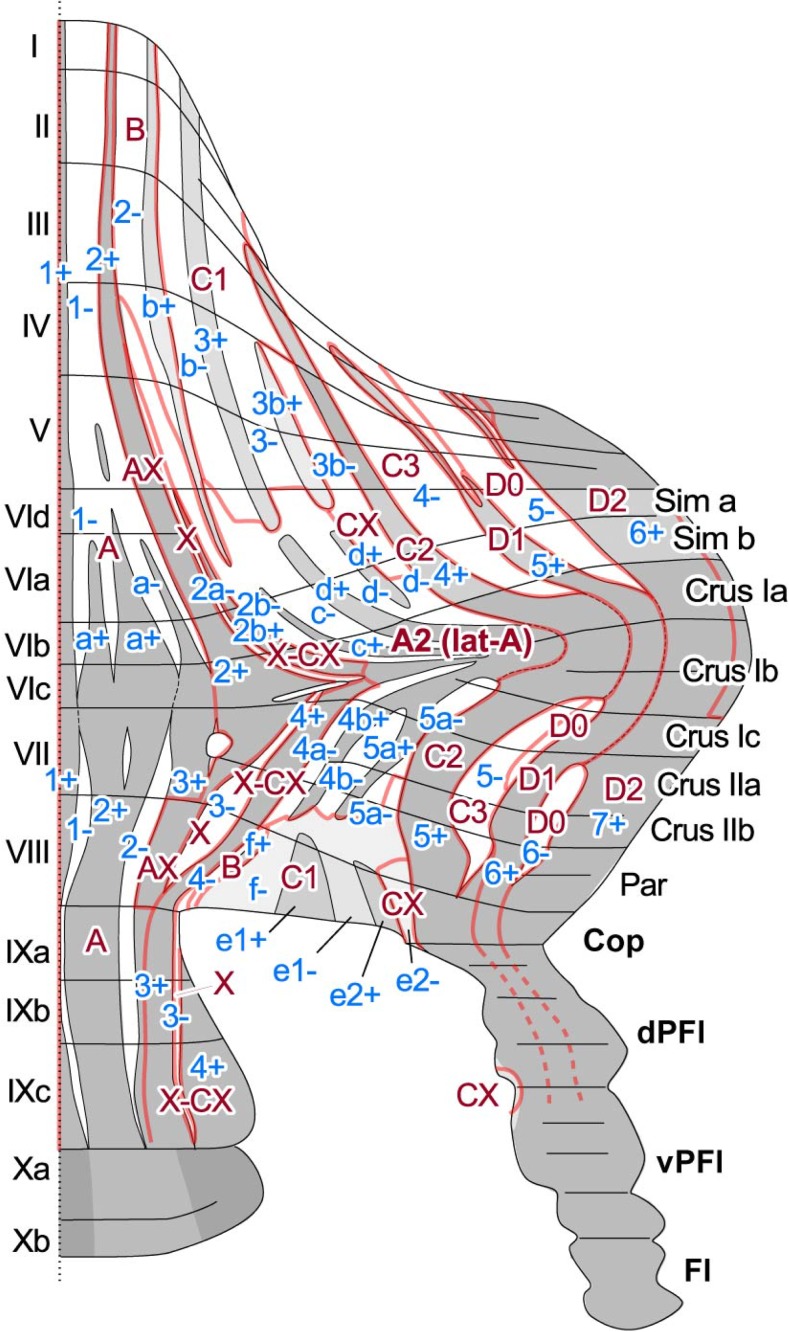
Schematic of positional correlation between zebrin II (aldolase C) striped pattern and the cerebellar module mapped on the unfolded rat cerebellar cortex in the rat. Based on Sugihara and Shinoda [10]

A clear immunostaining pattern with high contrast between negative-positive longitudinal stripes was reported with a monoclonal antibody that recognizes originally unidentified antigen “zebrin II” (ZII) [44], which was later identified as the isozyme of glycolytic enzyme aldolase C. ZII (aldolase C) expression pattern is clearly correlated with cerebellar modules. Conventional modules A, B, C1, C2, C3, D0, D1, D2 and later added modules such as X, CX, X-CX [45] are located in identified ZII expression stripes in the rat [10, 34, 46] (Fig. 3, Table 1). Therefore, the ZII-striped pattern is very useful as a landmark structure for the cerebellar modules. However, ZII+ stripes are less useful as a modular boundary marker in a few areas in which ZII+ stripes are neighboring with themselves, as well as in neighboring ZII− stripes. For example, B, C1, CX, and C3 modules, which are generally ZII−, are neighboring in the paravermal area in the anterior lobules and in lobule VIII (and its lateral extension copula pyramidis or copular part of the paramedian lobule). C2, D1, and D2 modules, which are generally ZII+, are neighboring in crus I and paraflocculus.

**Table 1:** Simplified correlation between the cerebellar module and zebrin stripes. This table is based on studies in the rat [6, 10, 34, 46, 47]. See Sugihara et al. [47] for a more detailed description

Module (cortical zone)	Zebrin II (aldolase C) stripe		Topographic connection	
	lobules I–VI	lobules VII–IX	CN	IO
A	1+, 1−, a+, a−	1+, 1−, 2+, 2−	MN	cMAO
AX	2+	3+	MN	cMAO
A2	c+, c−, d+, d−	4b+, 4b−,5a+, 5a−	DLP	cMAO
B	2−	4−	LVN	dDAO
X	2a−	3−	ICG	cMAO
CX	3b−	e2−	PIN	cMAO
X-CX	2b+	4+	PIN	DMCC
C1	b+, b−, 3+, 3−	f+, f−, e1+, e−	AIN	vDAO
C2	4+	5+	PIN	rMAO
C3	4−	5−	AIN	vDAO
D1	5+	6+	LN	vPO
D0	5−	6-	DLH	DM
D2	6+	7+	LN	dPO

### Expression of Other Molecules in Cerebellar Modules

Some molecules, such as excitatory amino acid transporter 4 (EAAT4) and phospholipase Cbeta3 (PLCβ 3), are expressed in the same striped pattern as ZII. Other molecules, such as PLCβ4, are expressed in a striped pattern that is completely complementary to the ZII pattern. Thus, the expression patterns of these molecules are correlated with cerebellar modules in a similar or complementary way to that of the ZII expression pattern.

Recently, the expression pattern of protocadherin 10 (Pcdh10) has been examined in the embryonic and postnatal mice [48]. This molecule is expressed strongly in four particular subareas in the embryonic cerebellum. In the later stages until adulthood, these subareas are integrated into the zonal organization of the cerebellar cortex. While the three medial Pcdh10-positive subareas are located within the A module and lateral A module in the adult cerebellar cortex, the most lateral Pcdh10-positive subarea (named “mid-lateral”) is transformed exclusively into the complete C2 module in the paravermis. Thus, Pcdh10 is a specific marker for the C2 module in the paravermal cerebellum.

### Visualization of the Modular Organization by the Molecular Expression Pattern

By labeling the molecule that is expressed in correlation with cerebellar modules, the morphological entity of cerebellar modules can be directly visualized, thereby facilitating analysis of the detailed spatial organization of modules. ZII stripes are generally shifted laterally in lobules VI–VII and crus I and negative stripes are absent in the apex of crus I. These characteristics of the ZII-striped pattern reconfirmed the proposed morphology of cerebellar modules in crus I, where modules are shifted laterally and C1, C3, or D0 modules are absent [46].

Module A, which covers nearly the whole vermis, is large. Lateral module A covers the paravermal area of simple lobule, crus I, crus II, and paramedian lobule. These modules contain both ZII+ and ZII− stripes. We proposed that within module A, the pattern of ZII stripes represent an organization of cerebellar compartments that is distinct in functional localization to some extent, and classified the stripes into three groups [10]. In other words, we proposed that the ZII-striped pattern within module A and lateral module A indicates submodular organization in these areas.

The modular organization makes an intricate complex in the paravermal cerebellar cortex. The composite of three main modules (C1, C2, and C3) and later-reported modules (X, CX, and X-CX) [45] has been confirmed in ZII stripes [47]. Within C1 module, several “lightly” ZII+ and ZII− stripes are recognized such as 3+ and 3b+ in the anterior lobe and e1+ and e2+ in lobule VIII. The Purkinje cells of these stripes are not as strongly labeled with ZII+ as the other zones, but nevertheless stand out within the ZII− stripes on either side of them. These lightly ZII+ stripes of the C1 module have specific topographic connections with slightly different areas in the cerebellar nuclei and the inferior olive [9]. Thus, these ZII stripes may represent a submodular organization as well.

### Experiments in Animal Models in Which Modules Are Visualized

Immunostaining of the cerebellar cortex after physiological recording or axonal labeling enables identification of the location of recording sites and axonal terminals into identified cerebellar modules. By this technique, synchronous complex spike activity in PCs within a module has been clarified [49]. Some different properties of PCs belonging to different modules have also become evident [50], as described in other sections of this article. Module-specific climbing and mossy fiber axonal projections have been revealed [10, 51].

Animals in which one of these molecules is visualized can be used in experiments of modules. We developed Aldoc-Venus mice in which mutated green fluorescent protein, Venus, is visualized in cells in which aldolase C (ZII) is expressed. The expression pattern of Venus accurately reproduces aldolase C expression. The striped pattern of aldolase C is not altered in Aldoc-Venus mice heterozygotes or homozygotes. Experiments about identified cerebellar modules are in progress by using aldoc-Venus heterozygous mice in vivo and in vitro. Tsutsumi et al. [52] used similar aldoc-tdTomato mice and recorded calcium signals, the rise of which is equivalent to a complex spike, from all PCs in multiple identified aldolase C stripes in the apex of crus II.

### Conclusion

Identification of the positional correlation between the cerebellar modules and molecular expression patterns has clarified the morphological entity of the cerebellar module. Labeling of these molecules facilitates studies of module-specific axonal connections, neuronal activities, and developmental mechanisms. Thus, although the mechanisms or functional consequences of module-related molecular expression have not been fully clarified, an understanding of the functional significance of cerebellar modules has been advanced recently.

## Optic Flow Modules in the Vestibulocerebellum of Pigeons (D.R. Wylie)

Self-motion of an organism through a world cluttered with visual stimuli results in “optic flow” across the entire retina [53]. This visual information is analyzed by retinal-recipient nuclei in the pretectum [54] and accessory optic system (AOS) [55], and reaches the vestibulocerebellum (VbC) via particular subnuclei in the inferior olive [56]. The VbC includes the flocculus, nodulus, and uvula, and is a site of visual-vestibular integration important for the generation of compensatory eye movements and the analysis of self-motion [57–59].

**Fig. 4:**
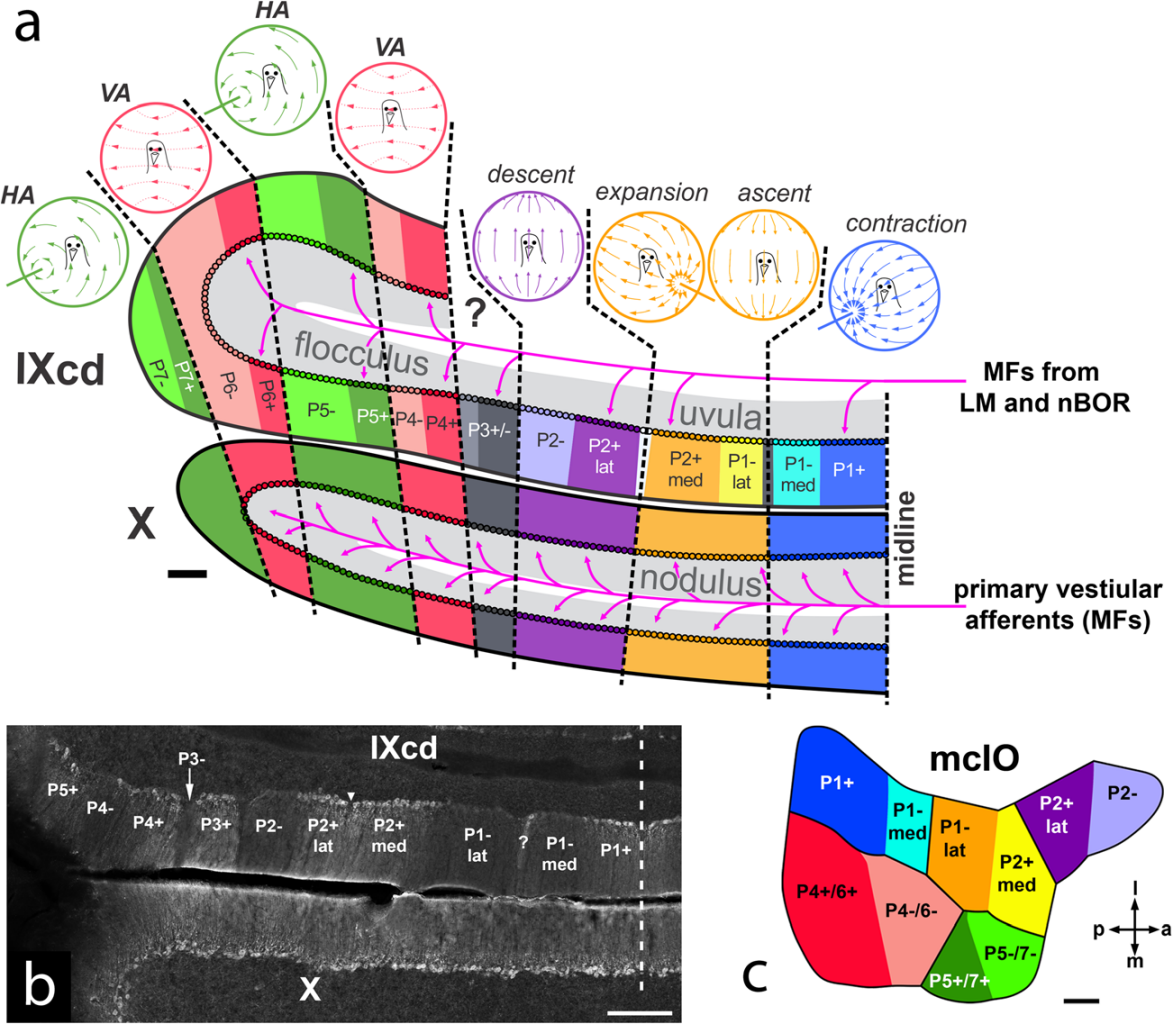
**a** Diagram of the optic flow modules in the pigeon vestibulocerebellum (VbC; folia IXcd and X) (based on data from [66–69]. The lateral half of the VbC is the flocculus, the medial half is the uvula (IXcd)/nodulus (X). Each module is represented by a depiction of the optic flowfield that maximally excites the complex spike activity (CSA) of the Purkinje cells (PCs). The ZII+ and ZII− stripes in IXcd are also indicated. (All PCs in X are uniformly ZII+). There are seven optic flow modules, each spanning a ZII+/− stripe pair (see text for details). P3+/− PCs do not respond to optic flow. The magenta arrows indicate the primary vestibular afferents, which project as mossy fibers (MFs) to X. Magenta arrows also show the optic flow MF inputs from the nucleus of the basal optic root (nBOR) and pretectal nucleus lentiformis mesencephali (LM) to the ZII+ stripes in IXcd. **b** Coronal section through ventral IXcd and dorsal X, showing the ZII expression. The inverted triangle indicates the “notch” where PCs are absent, and bisects the P2+ stripe in to medial and lateral halves (P2+med, P2+lat). The “?” indicates a ZII+ stripe, 1 to 3 PCs in width, which similarly divides the P1−stripe (P1−med, P1−lat). The vertical dashed line indicates the midline. **c** Dorsal view of the medial column of the inferior olive (mcIO) and is color-coded to match the ZII stripes in (**a**), to indicate the topography of the climbing fiber projections (based on data from [32, 33]). *a* anterior, *p* posterior, *m* medial, *l* lateral. Scale bars: 200 μm in (**a**), 300 μm in (**b**), 100 μm in (**c**)

In birds, where the cerebellum essentially appears as a vermis without hemispheres [60], the VbC includes folia IXcd and X [61]. The optic flow information to the VbC originates in the pretectal nucleus lentiformis mesencephali (LM) and the nucleus of the basal optic root (nBOR) of the AOS [62–65]. The pigeon VbC shows many aspects of the classic modular organization of the cerebellum [18] as shown in Fig. 4a. The complex spike activity (CSA) of Purkinje cells (PCs) in the pigeon VbC responds best to particular patterns of optic flow resulting from self-translation or self-rotation through space, and these PCs are organized into sagittal zones across folia IXcd and X. As in mammals, CSA in the flocculus is modulated by rotational optic flow about either the vertical axis (VA neurons) or an horizontal axis oriented 45° to the midline (HA neurons) [70–72]. In pigeon, there are two VA zones interdigitated with two HA zones [73]. In the uvula/nodulus, the CSA responds best to optic flow resulting from self-translation [66]. There are four response types organized into three sagittal zones. In the most medial zone, CSA responds best to optic flow resulting from translation backwards along an horizontal axis 45° to the midline such that there is a focus of contraction at 45° contralateral azimuth. Medial to this is a zone where the CSA responds best to optic flow resulting from either (i) forward translation along an horizontal axis 45° to the midline such that there is a focus of expansion at 45° ipsilateral azimuth, or (ii) upward translation along the vertical axis. Lateral to this is a zone where the CSA responds to the optic flow resulting from downward translation along the vertical axis [66]. A sagittal organization is also apparent with respect to the projection of PCs in the VbC: PCs in each of the optic flow zones project to particular regions in the vestibular and cerebellar nuclei [74–76]. Also, each of the optic flow zones receives climbing fiber (CF) input from particular regions of the medial column of the inferior olive (mcIO) [77, 78] (see also Fig. 4c).

A sagittal organization in IXcd is apparent with respect to the expression of Zebrin II (ZII; a.k.a. aldolase C [79]. As in mammals [44], ZII is heterogeneously expressed such that there are sagittal stripes of PCs exhibiting high ZII expression (ZII+) alternating with sagittal stripes of PCs that show little or no ZII expression (ZII−) [80]. In the VbC, there are seven stripe pairs (Fig. 4a). The most medial ZII− stripe, P1−, is bisected by a thin ZII+ stipe, such that P1− is divided into medial and lateral region (P1−med, P1−lat) (Fig. 4b). Similarly, the P2+ stripe is bisected by a notch that contains no PCs, effectively dividing the stripe in two halves (P2+med, P2+lat) (Fig. 4b). Using electrophysiological recordings combined with immunochemistry, we showed that the optic flow zones spans a ZII+/− stripe pair (Fig. 4a) [66, 67]. For example, the contraction zone spans P1+ and P1−med. As such, we consider that a ZII+/− pair represents a functional unit in the VbC, but what are the differences between the ZII+ and ZII− stripes within the unit? We have shown that they receive CF input from separate, but adjacent areas of the mcIO (Fig. 4c) [81, 82], and there is some suggestion that the ZII+ and ZII− PCs have differential projections [76]. We have some evidence that the CSA of ZII+ PCs shows a greater depth of modulation to optic flow stimuli, compared to the ZII− PCs within the same functional unit [83]. This applies if one compares ZII− and ZII+ PCs in IXcd, and if one compares the ZII− PCs in IXcd with the PCs in X (all ZII+). The depth of modulation of ZII+ PCs in IXcd is not different to that of PCs in X [83]. Moreover, the ZII+ and ZII− stripes likely receive different mossy fiber (MF) inputs. Both nBOR and LM project directly to IXcd as MFs [62, 63], and the majority (~ 85%) of these terminate adjacent to the ZII+ stripes [68] (Fig. 4a). It is not known if other MF afferents target the ZII− stripes.

Note that the optic flow zones span folia IXcd and X, but the ZII stripes do not. Rather, all the PCs in X are uniformly ZII+ [80]. Folia IXcd and X also differ with respect to MF inputs. The optic flow MFs from nBOR and LM mentioned above innervate IXcd, but not X. In contrast, there is a primary vestibular projection to folium X, but not IXcd [69] (see Fig. 4a).

In summary, the pigeon VbC contains optic flow modules that are sagittally oriented and span folia IXcd and X. The classic sagittal zonal organization is apparent with respect to PC response properties, CF inputs, and PC projections. However, there is clearly a transverse component to the modules as well, since IXcd and X receive discrete MF inputs carrying optic flow and vestibular information, respectively. Finally, the modules clearly contain subregions defined by neurochemistry, as each module encompasses a ZII+/− stripe pair. Whether this type of modular organization applies to other parts of the cerebellum, or the VbC in other vertebrate classes, remains unknown.

## The Cerebellum Quantum (R. Hawkes)

The modular nature of the cerebellar cortex suggests that it represents a map or family of maps, although what exactly is being “mapped” is less evident. The afferent topography is perhaps the simplest answer, in which case the map is fundamentally discontinuous in the sense that neighboring representations of body regions are neither anatomically nor physiologically continuous. What is the cerebellar “quantum”? In this context, the central idea is topographical equivalence: all cells in the “quantum” share a common chemistry, receive statistically identical inputs, project to the same target field(s), and have equivalent interneuron connectivity. Such a quantum would represent the smallest unitary processing unit.

Cerebellar modular architecture arose early in vertebrate evolution as the ground plan across birds and mammals is generally conserved. The largest cerebellar cortical compartments are the transverse zones (note that these are distinct from the sagittally oriented zones defined by olivocorticonuclear connectivity). In the mammalian vermis, four transverse zones are found in all species studied—the anterior zone (AZ), central zone (CZ), posterior zone (PZ), and the nodular zone (NZ) [84, 85] (in mouse a subdivision of the CZ has been identified—[86]: in birds, the ground plan has an additional transverse zone—the LZ [80]). Transverse zones evolve independently in response to different lifestyles (mosaic evolution). For example, in bats the echolocation centers in lobules VI/VII are accommodated by an expansion of the CZ—[87], and in the blind star-nosed mole, the CZ and NZ (visual receiving areas) are reduced and the trigeminal (star)-receiving areas (NZ and crus I/II) are expanded [88]. In sum, the cerebellar cortex comprises of the order ~ 10^1^ transverse zones: in a mouse each of ~ 10^4^ Purkinje cells (PCs).

Transverse zones are further divided into parasagittal stripes. How these stripes relate to the microzones identified by Oscarsson and his group [for review, see 88] is not certain: a tentative common framework is provided by the group of Voogd and Sugihara [10, 11, 13]. Stripes are discontinuous across transverse zone boundaries [85], suggesting that the earliest parcellation of the cerebellum during development is into transverse zones and subsequently these further subdivide into stripes. As is the case for zones, the number and variety of PC stripes is also not properly understood. The problem of how many stripes are present is exacerbated because many stripes revealed by ZII expression are, in fact, composite (e.g., heat shock protein-HSP25+/− subtypes within the ZII+ population [84]; PLCβ4+ sub-stripes within the ZII− population [89] etc.). As a consequence, the absolute number of stripes remains uncertain. Secondly, when molecular markers and mutant phenotypes are used in combination, some 10 PC subtypes can reliably be identified: this is likely an underestimate. By way of estimate, 5 transverse zones, each duplicated on either side of the midline, and 20 stripes per zone (based on connectivity plus chemistry) yields ~ 200 stripes per cerebellum, each comprising < 10^3^ PCs in the mouse. This is almost certainly an underestimate.

Stripes are further subdivided into strings of patches. For example, tactile receptive field mapping of trigeminal representations reveals an elaborate mosaic of somatosensory patches (so-called fractured somatotopy: [90–92], which in some cases have been shown to align with ZII+/− stripe boundaries [93, 94]. A complementary heterogeneity was also revealed by Garwicz et al. [43], further dividing microzones in the anterior paravermis (C3) of the cat into multiple rostrocaudal patches. Possible anatomical correlates of patches—blebs (e.g., [95] and expression markers, such as NOS [96] and dystrophin [97]—confirm an elaborate parcellation of the granular layer. The upshot is the dicing of stripes into several thousand functional patches, each comprising ~ 10^2^ PCs [98].

The cerebellar cortex is close to a pure feed-forward structure with little or no cross talk between neighboring stripes, so their proximity would seem irrelevant. However, this simplistic view may be wrong. Functional aggregates—limb inputs to the AZ, eye inputs to the flocculonodular lobes, trigeminal inputs to crus II, etc.—are found throughout the cerebellar cortex: indeed, this is the reality beneath the long-outdated idea of cerebellar homunculi. Such “neighborhoods” may be functionally critical due to MF data sharing via parallel fiber innervation.

So why does the cerebellum need a modular structure? We can suggest three reasons. First is the requirement for parallel processing. It is mandatory for the motor system to respond in a timely fashion and where there are so many degrees of freedom to control in an integrated manner serial processing is a non-starter. Hence, a highly parallel modular architecture has evolved to serve real-time motor control.

Secondly, the cerebellar cortex may exploit positional coding by assigning particular inputs to specific anatomical loci (limb inputs to stripes in the AZ; vibrissal inputs to patches in crus I/II, etc.). This re-encodes input modality as position (e.g., activation of a particular patch of crus II *ipso facto* implies ipsilateral vibrissal stimulation, etc.). Such positional coding ensures that minor sensory inputs are not dispersed and lost in the background noise. Positional coding also provides a substrate for the customization of the biochemistry once different patterns of gene expression are associated with particular zones, stripes, etc.; the door is open to regional specialization, tuning a stripe to its specific input/output requirements. Dozens of molecules are co-expressed differentially in stripes, both in the embryo and the adult. The question is—are the differences in stripe chemistry no more than genetic drift between paralogous PC populations or are they functionally significant? Evidence from several sources suggests that the latter option might be true (see the section by Chen and Ebner where the evidence is reviewed).

Thirdly, topographically equivalent quanta are a means to manage cerebellar signal-to-noise problems by exploiting the internal redundancy afforded by multiple, statistically identical PCs as a filter to generate a smoothed, more reliable output. The number of PCs needed—and hence the minimum quantum size—depends on how noisy each input is and how reliable the output needs to be.

In conclusion, the speculations above suggest that the cerebellar quantum is either a stripe (several hundred per cerebellum, each < 10^3^ PCs in mouse) or a patch (several thousand per cerebellum, each < 10^2^ PCs). This is not to imply that multiple quanta do not work in tandem to generate specific behaviors. First, perhaps cerebellar neighborhoods reflect a higher functional order—functionally related stripes/patches arrayed mediolaterally within a transverse zone and innervated by a common set of parallel fibers: stripes in the AZ processing forelimb signals also having access to hind limb information; vibrissal patches in crus I/II receiving contextual data about the lips and teeth, etc. Secondly, stripes may work as pairs—for example, ZII+/− stripe pairs in the pigeon NZ respond in concert to optic flow [79; and above]. Finally, multiple stripes may cooperate. Support for this view comes from data showing that networks of patches are linked by common MF inputs (see section by Spaeth et al.) and evidence that multiple stripes cooperate to control single muscles [99].

## Is the Micromodule the Minimal Functional Unit of Cerebellar Processing? (F. Bengtsson and H. Jörntell)

Based on anatomical and physiological mapping studies, there are some indications to support this view, but also some caveats that prevent us from drawing a definite conclusion.

First of all, one needs to define the terms used to describe functional units of the cerebellum. The terms modules and micromodules have historically been used in a confusing non-conformative way and here we try to disentangle the terminology. The relationship between a module and a micromodule is that a module is a sagittal zone of cerebellar cortex, the parts of the inferior olive (IO) that supplies that zone with climbing fibers (CFs), and the subdivision of the cerebellar nuclei (CN) that the sagittal zone sends its Purkinje cell (PC) axons to. A micromodule, or what members of our lab originally referred to as a microcomplex, consists of a microzone within the sagittal zone (each sagittal zone may contain several 10’s of microzones [100] and its associated subdivisions of the IO and CN [12, 40, 101]. The PCs of each microzone predominantly contact a small group of neurons in a specific CN subdivision, and here we refer to this set of neurons as a “micro-group.” Similarly, the PCs of each microzone receive CFs from a small part of a specific subdivision of the IO, and we refer to this set of IO neuron as a “micro-part” [101].

To date, there is no evidence to support that different PCs of the microzone control specific CN cells within the micro-group. Rather, individual PCs diverge extensively in their projection to the CN and each CN cell receives a wide convergence of PC inputs [102]. The lack of differential CN cell control within the micro-group is the rationale for assuming that it is acting as one unit, which consequently has one functional contribution. Caveat to this assumption is if separate PCs within the microzone are eventually shown to have differential control of these CNs, or if the mossy fibers that drive the CN cells [103] split this group into smaller functional units. Notably, there is a specific relationship between the receptive fields of the mossy fiber input and of the PC-mediated CF input to the individual CN cell [103], which suggests that the mossy fiber input to the CN cell is defined by learning and can therefore be expected to be homogenous for CN cells within the same micro-group. However, in the adult animal, the mossy fiber to CN plasticity do not seem highly active or easily induced [104], which of course does not contradict the possibility that it exists or that it might be highly active under development.

Although not included in the original concept of a micromodule, recent findings suggest that the inhibitory nucleo-olivary pathway should be included [105]. As the name suggests, the pathway originates in the CN and is under control of the PC output. A decreased PC firing will result in a disinhibition of the IO, thus forming a closed inhibitory feedback loop between the IO and the cerebellar circuit. The pathway seems to be zonally specific [106]. The spontaneous activity in the PCs is controlled by the level of IO input [107, 108]. Given that the assumption of a uniform micro-group of CN cells above applies, the total level of nucleo-olivary inhibition within a micromodule would be expected to be uniform and most of its PCs would have the same set point for their spontaneous firing activity. Different micromodules, however, may well have different levels of total nucleo-olivary inhibition and hence different levels of spontaneous PC activity. This scenario could work as an explanatory model for multiple reports that there are overall differences in the PC and CF activity between zebrin stripes [16, 17], as these appear to have a large degree of congruence with the functionally defined microzones [8].

The general idea that the modules of the cerebellum are functionally specific is supported by inactivation of specific areas of the IO, which results in functionally specific deficits in motor control [109]. The functional effects of the olivary inactivation can readily be explained as different modules predominantly project to different motor systems, i.e., vestibulospinal, tectospinal, reticulospinal, or rubrospinal systems as well as the corticospinal system [110]. For each micromodule, each CN micro-group can be expected to activate specific aspects of the function of the specific motor system for the module, which would be the cause of functional differences between micromodules. On the output side, each micro-group is divergent and contact strongly divergent upper motor neurons that in turn contact divergent spinal interneurons [111]. Yet, some center of gravity for which combinations of muscles each micromodule controls exists [99].

As every microzone has a specific function, assuming that it is the control of a specific set of muscles, for example, the PCs of the microzone will learn or potentiate specific mossy-/parallel fiber input that relates (sensory, motor, or sensorimotor) to the activation of that particular set of muscles. Depending on the specifics of a particular movement, different parallel fiber inputs will be active to a different degree and perhaps with a different temporal relationship to the CN output of the micromodule. Depending on the degree of correlation with the output effect of the CN group, subsets of parallel fiber inputs to PCs within a given microzone will be either potentiated or depressed. If the micromodule indeed is the minimal functional unit of the cerebellar circuitry, then the consequence is that the population of PCs in the microzone effectively is combined into one “super PC,” which operates with the same micro-group of CN neurons. The advantage of a super PC would be that it provides the possibility to sample a much higher total number of mossy fibers, from which the mossy fibers with the highest possible correlations with the micromodule activity functions can be selected, to the control function of the micromodule than a single PC alone would be capable of.

## Zonal Patterning of Mossy Fibers and Interneurons (A.M. Brown, E.P. Lackey, and R.V. Sillitoe)

Sagittal zones originate during early cerebellar development, and nearly all major cell types in the cerebellum respect the boundaries of zones [8, 112]. The zonal patterns of developing and adult Purkinje cells (PCs) have been extensively studied, but we are far from fully understanding how mossy fibers and the various types of interneurons are restricted within the zonal framework. This is an intriguing problem to consider from a circuit perspective because mossy fibers form mono- and di-synaptic connections to each class of interneurons in the cerebellar cortex.

Mossy fibers project from over two-dozen brainstem and spinal cord nuclei. Functionally similar mossy fibers terminate on granule cells within the same transverse domains in the cerebellar cortex. Within these transverse domains, mossy fiber terminal fields organize into parasagittal zones that have a reproducible anatomical relationship with olivo-cortico-nuclear modules. In contrast to climbing fibers, which terminate on just one or two contralateral zones of PCs, mossy fibers branch to terminate in multiple bilateral zones [113]. Furthermore, sensory information from different mossy fiber sources can converge onto single granule cells [114]. Cues derived from Purkinje cell clusters are thought to provide the organizational scaffold for the zonal distribution of both climbing fibers and mossy fibers. Purkinje cell clusters initially express transient parasagittal molecular markers as early as E14 in mice. Although Purkinje cell and climbing fiber patterning starts early, mossy fiber arrival in the cerebellum spans mid-embryonic and postnatal development [115]. This suggests that a protracted relationship might exist for module patterning to occur. Indeed, mossy fibers directly contact PCs through the second postnatal week in mice [116]. This idea is consistent with data showing that mossy fibers do not exhibit clear-cut zones until after birth [117]. Despite the clear heterogeneity of mossy fiber terminal field domains, their zones are generally broader and not as sharply defined as those of climbing fiber projections or the PCs [6]. Adding to this complexity is that mossy fiber receptive fields, mapped by recording granule cell responses to tactile stimuli, reveal multiple sensory representations of body parts in mosaic patches that form a “fractured somatotopy” [91, 93]. This complexity is mirrored in the organization of the mossy fiber targets, the granule cells. Granule cells are also restricted to transverse and parasagittal patterns of gene expression and these patterns are reflected by abnormalities detected after various experimental manipulations [96, 118–120]. Granule cell progenitors arise from the rhombic lip and proliferate in the external granular layer (EGL). Despite potential molecular differences in the progenitor populations, it is not clear how lineage influences the final patterning of granule cells. However, it seems that temporal mechanisms may distinguish broad transverse domains such that specific granule cells are fated to specific lobules [121]. It is also possible that interactions between the EGL progenitors and/or recently differentiated granule cells and Purkinje cell signals may direct parasagittal granule cell patterning. Between E11 and E14 in mice, cells arising from the rhombic lip travel to the EGL where, under the control of Purkinje cell signals, the EGL expands through progenitor proliferation. Granule cells must then traverse past the Purkinje cell dendrites and somata in order to reach what will become the granule cell layer [112]. During this time, Purkinje cell parasagittal zones could influence granule cell molecular phenotypes. It has also been suggested that mossy fibers might play an active role in patterning granule cell zones [120]. Interestingly, granule cell parallel fiber projections are also patterned relative to the Purkinje cell map (see section by Isope, Spaeth, and Valera).

Similar to granule cells, the excitatory unipolar brush cells also exhibit transverse and parasagittal zonal restriction. After they are born, unipolar brush cells migrate through the white matter en route to lobules IX and X, and by adult they localize to the granule cell layer [122]. Differential molecular expression distinguishes them into three subtypes, calretinin+, mGlrR1α+, and PLCβ4+, and mGlrR1α− and PLCβ4+, which all respect the parasagittal Purkinje cell zones [123]. There is compelling evidence to suggest that PCs have a large impact on the distribution of unipolar brush cells. For instance, unipolar brush cells lose their restriction to lobules IX and X when normal Purkinje cell patterning is disrupted by genetic lesions (e.g., via the deletion of *Ebf2*: [124]).

Much less is known about the zonal patterning of the inhibitory interneurons. Golgi cells, for example, exhibit molecular restriction in the anterior-posterior axis with some degree of morphological restriction to parasagittal zones. There are multiple molecular subtypes of Golgi cells, but so far, only the subtype expressing ZAC1 is known to be restricted to the posterior zone [125]. Golgi cell apical dendrites, which ascend into the molecular layer and contact parallel fibers, respect the borders of Purkinje cell parasagittal zones. Fewer than 3% of Golgi cell dendrites cross the borders of Purkinje cell zones and, though mechanisms have been suggested for this restriction, it is not clear how this relationship develops or is maintained [126].

Least is known about the patterning of basket and stellate cells in the molecular layer. Like Golgi cells, basket and stellate cells could exhibit a morphological restriction to zones wherein, particularly for basket cells and less so for stellate cells, their axons extend in the parasagittal plane. This may result in restriction of the inhibitory influence of the basket or stellate cells to specific zones [127, 128].

To achieve this restriction, it is possible that the parasagittal orientation of basket and stellate cell axons could have followed the spreading of Purkinje cell clusters into zones during cerebellar development. This argument is supported by the idea that modules might have their origins in the earliest stages of cerebellar development and therefore cells that are born later in cerebellar development, such as interneurons, develop within a circuit that is already committed to a zonal map. The outcome of these multicellular rearrangements plus the targeting of mossy fibers to the cerebellar input layer is thought to be modulation of Purkinje cell simple spikes via parallel fiber projections [129]. Both the frequency and regularity of simple spikes are dynamic during postnatal development and consistent with the maturation of parallel fiber synapses and establishment of mature Purkinje cell zonal expression patterns [129]. The maturation process of zones is mediated by spontaneous activity and sensory experience, which may intersect with genetic programs to integrate or sculpt mossy fibers into modules [112]. Ultimately, however, the formation and function of an operational module may depend on several factors including regional variations in Purkinje cell morphology, Purkinje cell packing density, granule cell packing density, neuronal soma size, intrinsic Purkinje cell firing properties, synaptic plasticity, the positions of mossy and climbing fiber synapses within their target layers, the distributions of the various cerebellar interneurons, and perhaps even glia [3].

## Modular Gene Expression Relates to Physiological Properties and Information Processing (M. Schonewille)

A wealth of anatomical and immunohistochemical data has revealed the modular organization of the cerebellum and its chemical landmarks, as described above. The efforts to understand the physiological and functional features of this organization have thus far not matched that. This section will discuss the progress made so far in analyzing the differences at the physiological level between modules in relation to the differential gene expression patterns.

### Module-Related Differences in Purkinje Cell Physiology

One of the first proteins to receive attention in this respect was excitatory amino acid transporter 4, EAAT4, which is expressed in Purkinje cells (PCs) in pattern similar to zebrin II (ZII) [130]. In ZII+ PCs, the synaptic transport current is several fold larger than in ZII− PCs [14]. Due to the absence of EAAT4, mGluR1 (metabotropic glutamate receptor 1) currents are larger and mGluR1-dependent long-term depression (LTD) is more likely to occur in ZII− PCs. Conversely, long-term potentiation (LTP) could be induced selectively in patches of ZII+ PCs using high-frequency stimulation of parallel fibers in vivo [15], which will be described in the next section. Interestingly, in another dataset, the climbing fiber evoked EPSCs and complex spikes in ZII+ PCs were found to be larger despite the presence of more EAAT4, suggesting that climbing fibers also participate in the modular differentiation [131].

**Fig. 5:**
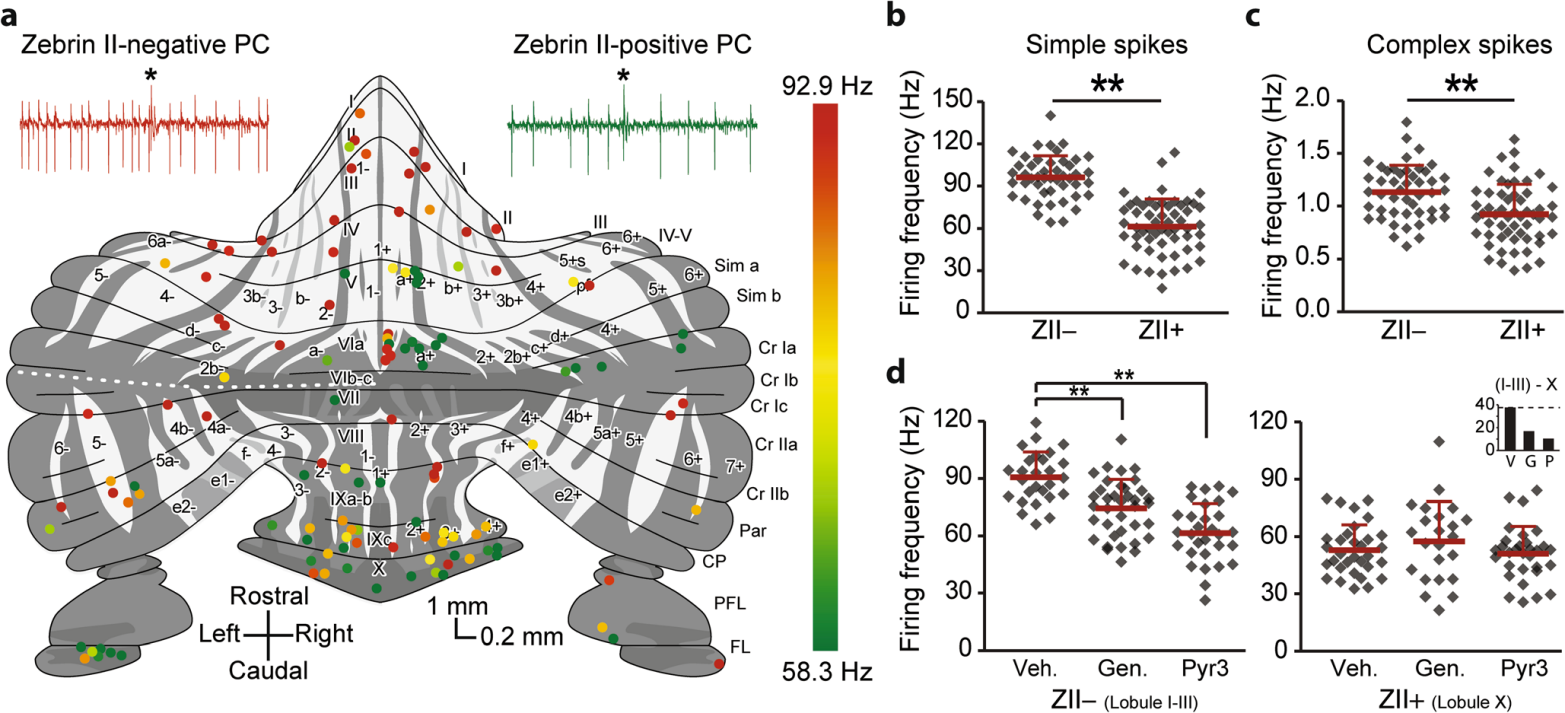
Physiological difference between zebrin-identified cerebellar modules. **a** Schematic drawing of unfolded cerebellar surface, adapted from [66–69], depicting post-mortem immunohistochemically determined recording locations of PC, with color-coded simple spike firing rate. Note the higher firing rate in ZII− PCs and the consistent presence of the difference, even in nearby pairs. **b** Summary of (**a**) demonstrating the significant difference in average simple spike firing rate between ZII+ and ZII− PCs, recorded in vivo. **c** Complex spike firing rates show a similar difference, with higher firing rates in ZII- than in ZII+ PCs. **d** Pharmacological block of TRPC3 with two difference blockers, genestein, and pyr3, selectively affects PC simple spike activity in ZII− PCs, indicating the contribution of TRPC3 to creating this difference

Not only plasticity, but also the most basic physiological cell property, firing rate, appeared to differ between cerebellar regions in vitro [132]. Indeed, in vivo, ZII− PCs in mice fire simple spikes at ~ 95 Hz, while ZII+ PCs on average fire at ~ 60 Hz during quiet wakefulness [17, 133] (Fig. 5a, b). This difference in firing rate is largely intrinsic to PCs and could be significantly reduced by blocking TRPC3 [17] (Fig. 5d, e). TRP channels are known to be the effector channels of mGluR1 [cf. 87, 134] and are part of a pathway including PLC, PKC, and IP3R1, which all have zebrin-related expression patterns. Similar experiments comparing ZII− and ZII+ areas in anesthetized rats confirmed the higher simple spike firing rate in ZII− PCs [16]. In this study, Xiao et al. observed a higher coefficient of variation (CV) for simple spikes in ZII− PCs. However, the use of anesthetics affects the regularity of PC firing [135], potentially explaining why the opposite result, higher regularity in ZII+ PCs, was found in awake mice [133]. Common finding in both studies is that some variations in other parameters are not related to zebrin patterning, suggesting further heterogeneity in PCs [50, 133, 136]. Overall, these results confirm a module-related differentiation of PCs, the sole output of the cerebellar cortex.

Climbing fiber input from the IO affects simple spike activity, both on longer and shorter timescales [137, 138]. In anesthetized rats, the impact of a complex spike was similar in both ZII+ and ZII−, but the effects were more prominent in ZII+ PCs [50]. In mice during quiet wakefulness, the effects appear to be related to the cerebellar modules. ZII+ PCs display changes in all directions, while ZII− PCs only show suppression or no change [17]. When TRPC3 is blocked, this restriction is removed and ZII− PCs show all types as well, suggesting that TRPC3 is also involve in post-complex spike effects on simple spikes [17].

### Module-Related Differences in Other Parts of the Olivocerebellar Circuit

Other parts of the olivocerebellar circuit also show zebrin-related differences. Complex spikes directly reflect the activity in the inferior olive. Theoretically, the higher simple spike rate in ZII− modules should provide stronger inhibition of the CN [139, 140], which would then disinhibit the IO [141–144], although the effects of this inhibition appear more complex [145, 146]. The prediction holds, as the complex spike rate is indeed higher in ZII− PC in awake mice [17] (Fig. 5c), although this was not confirmed in anesthetized rats [16]. Traditionally, the complex spike was considered to be an all-or-none phenomenon with a fixed underlying composition [147, 148], but there are functionally relevant temporal and spatial variations in its properties and consequences [149–151]. Some variations can be linked to the zebrin-based subdivision: the number of spikelets, for instance, correlates selectively in ZII− PCs with the simple spike firing rate, in rat [50]. The absence of this correlation in monkeys [152] could be due to species differences or related to the population with mixed zebrin identity. Together, these data suggest that the differentiation of physiological activity is present in at least two out of three nodes in the olivocerebellar circuit.

The question remains if the differentiation underlies fundamental differences in information processing and ultimately in function. The higher firing rate and preference for LTD [14] in ZII− PCs [16, 17] versus the lower rate with preferred LTP [15] in ZII+ PCs suggest this is indeed the case. In fact, some experimental evidence is in line with this concept. Eyeblink conditioning has been linked to ZII− PCs [153] that have a high resting rate, which is suppressed during the conditional blink [154–156]. In contrast, compensatory eye movement adaptation depends on ZII+ PCs in the flocculus (see, e.g., Fig. 3) that have a low resting rate and show potentiated activity during the adapted response [157].

Taken together, the current literature demonstrates that two out of three elements in the olivocerebellar circuit, the inferior olivary neurons and PCs, have distinct physiological properties that correlate with the zebrin-identified cerebellar modules. The differences are present at the level of cellular activity and interaction between inputs, both in the form of direct interactions and prolonged plastic changes. Future experiment should clarify the differentiation at the level of the cerebellar nuclei and determine the computational and ultimately functional relevance of this differentiation.

## Physiological Correlates of Zebrin II Parasagittal Zones (G. Chen and T.J. Ebner)

As detailed in the contributions to this consensus paper by Voogd, Sugihara, and Hawkes, a dominant feature of the cerebellum is its longitudinal architecture as defined by the parasagittal organization of its afferent and efferent projections and by the molecular compartmentalization of these parasagittal zones (see Figs. 2 and 3). Although highlighted by the expression of zebrin II/aldolase C (ZII), the parasagittal organization involves a host of other molecules, expressed on PCs in either a ZII+ or ZII− banding pattern of stripes [8, 158] [8, 158]. Importantly, many of these molecules control neuronal excitability, for example EAAT4 and mGluR1 subtypes.

The contribution by Schonewille describes recent studies on the differential firing characteristics of PCs in ZII+/− stripes, with the key observations that the spontaneous simple spike and complex spike firing rates are higher in ZII− than in ZII+ stripes (see Fig. 5 and Table 2) [16, 17]. Several of the firing differences are intrinsic to PCs as they persist when synaptic inputs are blocked, either pharmacologically or genetically [17]. The mGluR1 signaling pathway associated with ZII− PCs plays a role. However, neither EAAT4 nor aldolase C contributes to the intrinsic differences in firing rates, both of which are expressed in a ZII+ pattern. Building on the Schonewille review, this section focuses on two additional aspects of the physiological properties of ZII+/− stripes: responses to afferent inputs and synaptic plasticity.

**Table 2:** Functional difference between zebrin banding architectures

	Zebrin II+	Zebrin II−
Spatial pattern of activation	1. Parasagittal bands evoked by peripheral and inferior olive stimulation [159, 160] 2.Off-beam inhibitory bands evoked by PF stimulation [128] 3. mGluR1 mediated long latency patches by PF stimulation [15]	1. Less off-beam inhibition [128] 2. Peripheral stimulation evoked patches between EAAT4 bands [161]
CF-PC synaptic transmission	More glutamate released per CF action potential and longer EPSC [131]	
PC firing properties	1. Lower SS and CS firing rates [17] 2. Greater SS firing variability [16] 3. Higher incidence of SS suppression and oscillations following CS [17] 4. SS firing correlates with CS spikelets [50]	1. Higher SS and CS firing rates [16, 17] 2. More regular SS firing [16] 3. Greater relative SS pause following CS [16, 50]
Synaptic plasticity	1. No LTD [14] 2. LTP of mGluR1 mediated long latency patches [15, 162]	1. Robust LTD [14]

### Zebrin II+/− Stripes Respond Differentially to Various Inputs

Spinocerebellar and olivocerebellar afferent pathways activate parasagittally oriented responses in the cerebellar cortex [94, 159, 163, 164]. Simultaneous recordings reveal that climbing fiber input activates PCs in parasagittal zones with a rhythmicity of 6–10 Hz [165–167]. Optical imaging shows that inferior olive or peripheral stimulation evokes a marked parasagittal banding pattern that aligns precisely with the underlying ZII+ stripes (Table 2) [159, 160]. The bands are primarily due to climbing fiber input as they are optimally activated by 6–10 Hz peripheral stimuli and blocked by silencing the inferior olive. Two-photon imaging examining the relationship between ZII expression and synchrony at the single cell level observed that greater complex spike synchrony occurs among neighboring ZII+ or ZII− PCs but not across these two populations [52]. However, the stripes are not static, as sensory input increases the synchrony across ZII+/− boundaries in the awake animal.

Several factors contribute to the parasagittal responses including differences in (1) topography of climbing fiber and mossy fiber inputs to the cerebellar cortex and (2) intrinsic properties of the afferents, PCs, and molecular layer interneurons. Here, we concentrate on the intrinsic properties. Climbing fiber inputs to ZII+ stripes release more glutamate and generate larger, longer-duration AMPA-mediated excitatory currents in PCs than in ZII− stripes (Table 2) [131]. These differences in climbing fiber responses are largely presynaptic in origin and due to a larger pool of release competent vesicles and enhanced multi-vesicular release. In addition to the differences in climbing fiber afferents, the molecular specialization of PCs contributes to the parasagittal response pattern. In Crus II, the patch-like responses to peripheral stimuli are closely aligned to bands that express lower levels of EAAT4 (Table 2) [161], suggesting that PC responsiveness is controlled by the degree of glutamate uptake. Differences in EAAT4 expression also contribute to whether mossy fiber input evokes beam-like or patch-like responses [161]. Furthermore, several of the differences in PC simple spike firing, including the greater kurtosis and positive skewness in ZII stripes, appear input-driven [50].

Parallel fibers (PFs), the bifurcated axons of granule cells in the molecular layer, extend for 3–5 mm along the long axis of a folium and make glutamatergic synapses with the dendrites of PCs and cerebellar interneurons. In many folia, PFs cross several parasagittal bands and it is generally assumed that PFs provide for relatively uniform, short-latency activation of their postsynaptic targets [168]. However, PCs in ZII+/− stripes respond differently to PF input (Table 2) [15, 128]. Flavoprotein and Ca^2+^ imaging show that PF stimulation evokes an excitatory on-beam response and a compartmentalized off-beam response consisting of parasagittal bands of decreased fluorescence [128]. These off-beam bands are in register with ZII+ stripes, blocked by GABA_A_ receptor antagonists, associated with inhibition of PCs and spatially modulate the response to peripheral inputs. Also, PF stimulation evokes mGluR1-dependent patches of increased fluorescence at very long latencies that are aligned with ZII+ stripes [15, 162]. Therefore, the ZII striping pattern modulates the responses to both peripheral and PF inputs.

### Zebrin II+/− Purkinje Cells Have Different Synaptic Plasticity

PCs in Z+/− stripes exhibit different levels of synaptic plasticity. Conjunctive stimulation of PF and climbing fiber inputs results in long-term depression (LTD) of PF synapses on PCs and LTD plays important roles in motor learning [169]. Intriguingly, LTD was not observed in lobule X that uniformly expresses ZII+ and a high level of EAAT4 (Table 2) [14]. Conversely, robust LTD occurs in lobule III that is primarily ZII− and has low levels of EAAT4. The zonal expression patterns of mGluR subtypes and EAAT4 act to reduce the mGluR1 responses in PCs and prevent the induction of LTD. Increased EAAT4 levels in ZII+ stripes enable faster clearance and limit glutamate diffusion [14, 131]. Also, long-term potentiation (LTP) of PF synapses on PCs can be evoked by several induction protocols [15, 170, 171]. While less well studied than LTD, one difference has been reported for the LTP of the long-latency patches evoked by PF stimulation [15]. In response to theta burst PF stimulation, the long-latency patches, which are aligned with ZII+ bands, show dramatic LTP that is both mGluR1 and PLCβ dependent [15, 162].

In summary, the parasagittal compartmentalization of PCs has strong counterparts in physiological function that includes differential responsiveness to inputs, intrinsic excitability, and synaptic plasticity. Of the possible PC signaling pathways, to date mGluR1s and EAAT4 have been shown to have the more prominent roles in shaping the physiological differences between ZII+/− stripes. However, lacking is a unifying hypothesis on what functions these intrinsic differences play in the cerebellum’s role in motor and non-motor functioning. What is needed are studies that identify the specialized information processing occurring in ZII+/− stripes during behavior and determine how those unique computations are used by the cerebellum.

## Toward a Description of the Functional Modular Organization of the Cerebellar Cortex (P. Isope, L. Spaeth, and A. Valera)

In this section, we will review how the interplay between the mossy-fiber (MF)/granule cell (GC)/Purkinje cell (PC) pathway and the olivo-cerebellar system determines a functional modular organization.

### Cerebellar Modules and MF Projections in the Cerebellar Cortex

Previous sections have established that cerebellar modules are essentially defined by the olivo-cerebellar loop. The cerebellar cortex is divided into a large number of parasagittal bands subdivided into 100–200-μm-wide “microzones” that contain PCs excited by CFs driven by the same peripheral inputs [11, 172–174]. Since the stimulation of restricted areas of the cerebellar cortex [175] or the cerebellar nuclei [176] evokes movements of the receptive fields from which sensory inputs originated, a segregated information processing is potentially maintained throughout the olivo-cerebellar system [12]. Furthermore, CFs gate long-term plasticity induction at the GC-PC synapses [177, 178], a major site for information storage in the cerebellum suggesting that microzones may work as paralleled processing units for motor learning [179]. However, a pure parallel processing is unlikely for several reasons. First, individual microzones project on different targets (see section by Aoki and Ruigrok) suggesting that a given body area receives information from many microzones. Secondly, the anatomical organization of the MF-GC-PC pathway [2], which convey the afferent copy of the motor command or the planned action (from the cerebral motor, premotor and frontal cortices via the pontine nuclei) and the current status of the body (from the spinal cord), compromises a strict parallel information processing [180–184]. Indeed, MFs project onto GCs that contact hundreds of PCs in the same lobule via their parallel fibers (PFs) [185, 186] and transmit the information to several microzones in the transverse plane. Also, a wealth of tracing studies have demonstrated that in many areas of the cerebellum, MFs send a high number of collaterals in the GC layer both in the transverse (e.g., projection from the lateral reticular nucleus, dorsal column nuclei, and pontine nuclei) [187, 188] and in the sagittal orientation (e.g., collaterals of the dorsal spino-cerebellar tract targeting both lobule I–III and VIII) [189], suggesting that a given input is heavily redundant in the cerebellar cortex. Moreover, in a given GC layer area, MF from different sources overlap even at the level of individual GCs [114, 190, 191]. For example, in the anterior lobe of the vermis, MF inputs from the dorsal spino-cerebellar tract (hindlimb), the external cuneate (forelimb/shoulder), the cervix (forelimb, neck, and upper trunk), and from the pontine nuclei, overlap [113, 123]. The MF-GC pathway is therefore highly divergent and favors combinatorial processing and pattern discrimination as suggested by Marr and Albus, and Ito [2, 192, 193]. This organization must promote the communication between cortical microzones via the PFs and might determine a coordinated PC output to the cerebellar nuclei. Because adjacent microzones can express different zebrin markers leading to specific physiological properties and/or plasticity (see sections by Hawkes, Chen and Ebner, Schonewille), we can postulate that PFs multiplex modules are involved in specific tasks.

### The Functional Cortical Module: a Spatial Code

**Fig. 6:**
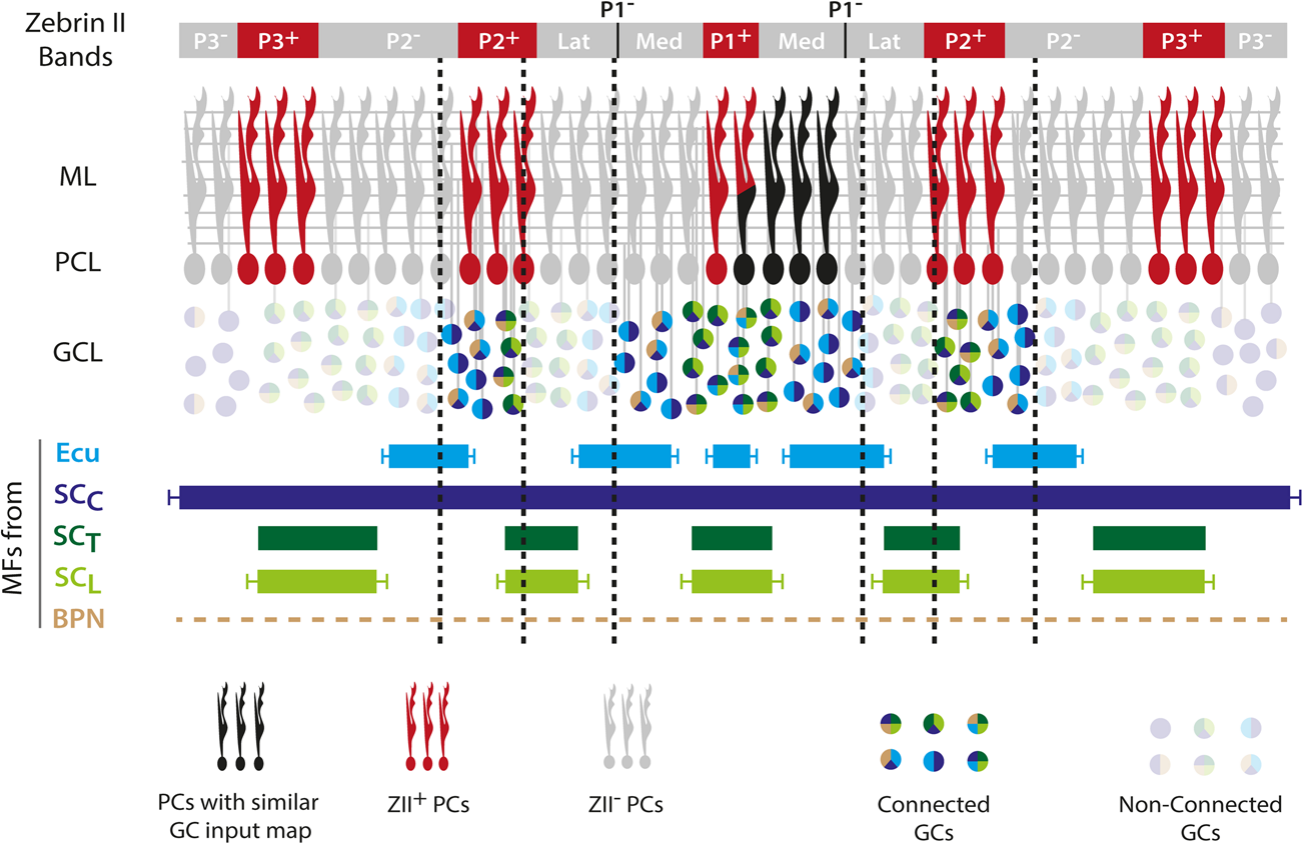
An example of a functional Purkinje cell module in the lobule III–IV of the cerebellar cortex. GC clusters belonging to different microzones (identified by the zebrin band pattern in red and gray) communicate with specific groups of PCs (one example in black). In this example, a group of PCs (120 μm width spanning P1− and P1+ zebrin stripes) close to the midline receives GC inputs from ipsilateral and contralateral P2+, ipsilateral and contralateral median P1− and P1+ microzones. This organization is conserved across mice. Each GC cluster receives specific MF inputs from different precerebellar nuclei and modalities (identified by the color in the GC pie chart). MFs projections in the GCL are complex and redundant. The other GCs remain silent or unconnected (shaded pie chart). This functional module does not necessarily fit with the anatomical boundaries given by the CF and MF inputs. *ML* molecular layer, *PCL* Purkinje cell layer, *GCL* granule cell layer, *MFs* Mossy fibers, *Ecu* external cuneate, *SCL* lumbar part of the spinocerebellar tract, *SCT* thoracic part of the spinocerebellar tract, *SCC* cervical part of the spinocerebellar tract, *BPN* basal pontine nuclei, *CFs* climbing fibers, *MFs* mossy fibers, *PCs* Purkinje cells, *GCs* granule cells. Adapted from [113, 199]

Paradoxically, although MF projections are redundant and overlapping, several groups demonstrated that microzones have the same MF and CF receptive fields (i.e., from the same body area) [174, 194, 195], some of them even suggested that local MF inputs represent the major and unique input to PCs through the ascending GC axon [195]. On the contrary, in vitro and in vivo studies have identified dense and localized distant synaptic connections between PCs from a given microzone and the MF-GC pathway belonging to another microzone [161, 196–199]. In fact, all these results can be reconciled by the fact that 85% of the GC-PC synapses are silent [197], that a limited number of GC layer sites are heavily connected to a given microzone [196, 199], and that PCs are always contacted by local GCs as a non-conditional input [195, 199, 200]. Furthermore, zebrin stripe identity may also account for local vs. distant communication [161] through the level of glutamate transporter (see section by Chen and Ebner). Strikingly, in the anterior vermis, the functional synaptic organization between microzones at the GC-PC and GC-molecular interneurons (MLIs) synapses is conserved among mice [199]. Bands of neighboring PCs (60 to 120 μm width) display the same GC input maps with local and distant clusters of GCs densely connected (dense clusters of GCs have been also observed recently in vivo [201–203]). These conserved networks define functional modules (with super PCs as proposed in the section by Bengtsson and Jörntell) in the cerebellar cortex that do not necessary match anatomical CF and MF input boundaries and zebrin stripes **(**Fig. 6). Activity-dependent mechanisms can also modify these maps through the awakening or the depression of GC-PC synapses [199]. Therefore, functional modules adapt under behavioral control. Altogether, these findings highlight the specificity of the MF/GC/PC functional maps and the communication between identified microzones, which define a spatial code of related modules. In this context, we should then refine our definition of the cerebellar modules and consider microzones as the anatomical modules while specific combinations of GC, MLI, and PC groups distributed in several location of the cerebellar cortex define the functional correlate of these modules **(**Fig. 6**)**.

### The Functional Cortical Module: a Temporal Code

This functional spatial modular organization of the MF/GC/PC pathway might influence temporal coding in PCs. Indeed, the temporal organization of the MF discharges, which span a wide range of frequencies in different cerebellar lobules, strongly influences the output of the GC layer [190, 204–210]. During high-frequency bursts of MF inputs, temporal summation may favor an explosive integration and a high signal-to-noise ratio as GCs may be excited by only one or two different MFs [205, 209]. Several studies have also demonstrated that burst of stimulation at frequency up to a kHz are reliably decoded both at the MF-GC and at the GC-PC synapses [211–213]. At lower frequencies, for example in the vestibular cerebellum, the combination of several sources of MFs with pathway-specific short-term synaptic plasticity leads to a precise temporal code in targeted GCs [190]. Finally, GC discharges in the clustered GC groups are gated by the Golgi cells through a double mechanism: (1) a feedback inhibition through the ascending axon of the GCs [214] as a gain control mechanism and (2) a feed-forward inhibition through the MF-Golgi cell pathway that increases the saliency of the MF signaling by improving the reproducibility of the GC firing [207]. Therefore, there is strong evidence that clusters of GCs at a given location respond to a specific spatiotemporal configuration of MF inputs. Since GC clusters are specifically connected to bands of PCs, this arrangement may control and broadly synchronize identified modules.

In conclusion, recent advances have described adaptive functional modular information processing in the cerebellar cortex. Combinations of MF inputs that activate local or distant clusters of GC in specific sequences are selected by groups of neighboring PCs that define a functional cortical module (Fig. 6). The selection process is gated by the CF inputs targeting these PCs. The dynamical interactions between these functional modules determine the collective pattern of discharge at the PC output layer and define a population code of a given behavioral component [215].

### Output of Cerebellar Modules (S. Aoki and T.J.H. Ruigrok)

Although exhaustive research has provided many details of the anatomical, chemical, and physiological characteristics of cerebellar modules, it is still not clear how these modules contribute to improved learning and execution of all kinds of movement [3, 6, 12]. In particular, it is not simple to envision the precise function of a given module and to understand how, or to what extent, different modules operate independently or need to cooperate for optimal behavioral output. If, as is generally assumed, individual modules can be seen as operational entities, each module is expected to participate in a different functional aspect of cerebellar control [4, 12, 216]. Indeed, the output of every cerebellar cortical zone of Purkinje cells (PCs), or microzone, by way of its converging projections to a specific location in the cerebellar or vestibular nuclei, is subsequently fed into a specific array of different nuclei in the brainstem and diencephalon [217, 218]. However, tracing studies also indicate that projection patterns of different cerebellar nuclei can have the same target area or that different regions of the same cerebellar nucleus can select rather different goals [218, 219].

Furthermore, although it seems quite clear that the nucleo-olivary projection stems from a class of small GABAergic cerebellar nuclear neurons that are mostly intermingled with the other projection neurons [220, 221], the latter neurons project and collateralize to functionally rather diverse areas ranging from upper spinal cord to diencephalon [222, 223]. From these projection targets, it can be surmised that an important part of the cerebellar output is fed back into the cerebellum by way of a multitude of feedback circuits. Not only can cerebellar output directly impact the cerebellar cortex by way of nucleo-cortical collaterals [224–226], but reverberating loops are also found by projections to precerebellar centers such as the magnocellular red nucleus (involving the cerebellar nuclei), the basal pontine nuclei (involving mostly the cerebellar cortex), or the reticulotegmental nucleus of the pons (involving both nuclei and cerebellar cortex) [227–230]. Moreover, both direct (i.e., nucleo-olivary projection) and indirect circuits (involving midbrain nuclei) link cerebellar output to the inferior olive [141, 145, 231]. Different modules make use of different or different combinations of these feedback reentrance circuits. For example, the GABAergic output of all modules is directed to their respective part of the inferior olivary complex [141, 232], whereas especially the ZII+ stripes seem to make use of an excitatory olivary connection by way of midbrain nuclei such as the parvicellular red nucleus and the nuclei of Bechterew and Darkschewitsch [13]. More elaborate circuitry involving the thalamus, cerebral cortex, and basal pontine nuclei also seems to operate [233, 234]. Between all these different targets, such as thalamus and medullary reticular formation, profuse collateralization of nuclear efferents is observed [222, 223]. In this respect, it is hard to understand how this system of diverging and partly converging connections is being used in an integrated way to result in coordinated learning and execution of movements, cognitive and affective behavior, or visceral functions [4, 235].

Best-known examples of the functional properties of cerebellar modules are illustrated by the ample bulk of research studying the adaptive control of reflexive eye and head movements. Here, individual floccular modules control movements around a particular visual axis [82, 236, 237]. However, it should be noted that also in the vestibulocerebellum, several non-adjacent zones seem to be present with basically the same function [71]. Furthermore, apart from floccular control of eye movements, cerebellar control of saccades stems from the flocculus, quite different cerebellar regions control saccades and voluntary eye movements [238, 239]. This would inevitably result in a multimodular control of individual eye muscles, with each module dealing with a certain aspect of control.

**Fig. 7:**
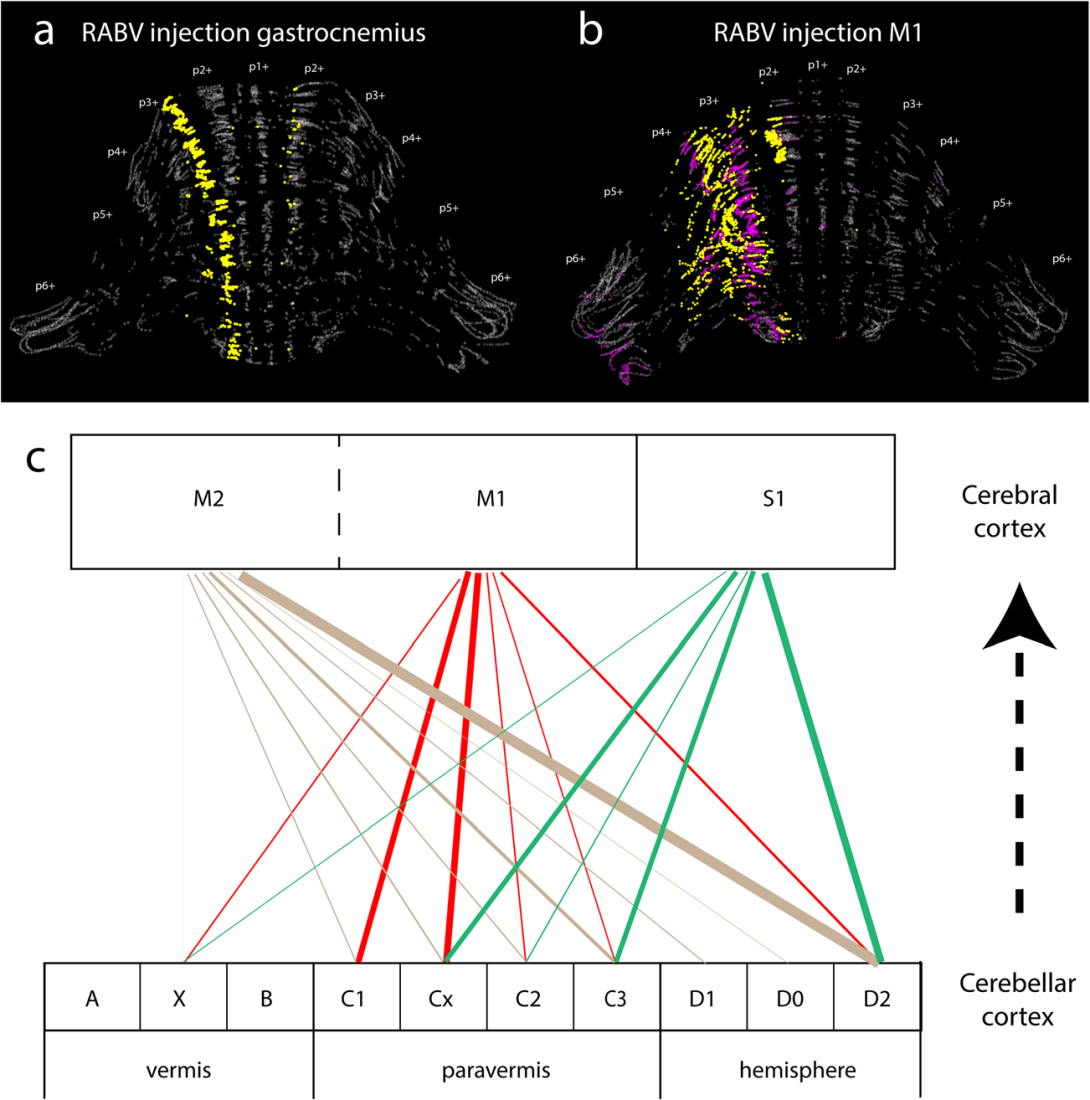
Multiple modules collaborate in sensorimotor processing. **a** Superimposed stack of plots of 10 serial (1 out of 4), 40-μm-thick sections showing RABV−/ZII+ Purkinje cells (gray), RABV+/ZII− (yellow), and RABV+/ZII+ (magenta) Purkinje cells in the rat anterior lobe 120 h after injection of RABV in the gastrocnemius muscle (case 1010). Note that a prominent band of RABV+/ZII− Purkinje cells is seen between the P1+ and P2+ zebrin stripes, which mostly is territory of the B zone [240]. The main zebrin+ stripes (p1+ to p6+) are indicated. **b** Similar superimposed stack of 12 plotted sections 70 h after injection in M1 (case 1151). Note that three separated clusters of RABV infected Purkinje cells are recognized: a vermal one just lateral of P2+, a large paravermal cluster that encompasses P3+, P3−, P4+, P4− zebrin stripes and a hemispheral cluster lateral within the P6+ strip. **c** Diagram showing multimodular impact of cerebellar zones on three sensorimotor regions. Line thickness is shown relative to 100% of total number of RABV labeled Purkinje cells in the anterior part of the cerebellum (modified after [241]). Modular identity is inferred from its relation with the zebrin pattern [11, 99]

This aspect was also demonstrated in a study in rat in which several hind- or forelimb muscles were injected with a transneuronally transported rabies virus (RABV). Retrograde RABV infection of PCs occurred by way of initial infection of reticulospinal, vestibulospinal, and rubrospinal pathways. In this way, it was shown that several cerebellar modules contribute to the control of individual muscles. For example, injection of the anterior tibial muscle of the rat initially resulted in infection of PCs that control the lateral vestibular nucleus (B zone, Fig. 7a), but subsequent zonal infection of PCs that contact the medial cerebellar nucleus, anterior interposed nucleus, and dorsolateral hump established that all these zones (and modules) all seem to be involved in the control of this muscle. Injecting its antagonist (e.g., anterior tibial muscle), to some extent, resulted in infection of the same PCs, although differences were also observed [99]. As the transneuronal transport of RABV depends on the number (and strength) of the synaptic steps within a particular pathway, the cerebellar impact on other routes than the rubro-, vestibulo-, and reticulospinal pathways, such as the corticospinal pathways, on these muscles could not be studied [99].

For this reason, we have recently made RABV injections in different places of the sensorimotor cortex of the rat, with the intention of studying the cerebellar modular involvement in processes that take place in the cerebral cortex. The survival time was carefully chosen to not exceed third-order labeling. In this way, after first-order RABV infection of the thalamic relay, and second-order infection of cerebellar nuclear neurons, third-order RABV infection of PCs was identified in various places of the cerebellar cortex (Fig. 7b). The main result here was that injections centered in either primary (M1), or secondary (M2) motor cortex or primary somatosensory cortex (S1), all resulted in multiple, zonally arranged, aggregates of RABV-infected PCs [241]. As these aggregates were observed in vermal, paravermal, and hemispheral regions of the cerebellum, it was concluded that the information from different cerebellar modules converges to a particular cerebral domain, suggesting that cerebellar modules cooperate not only through controlling several descending bulbospinal systems but also through interactively impacting cortical sensorimotor processing (Fig. 7c).

## The Cerebellar A Module and Emotional Behavior (C. Lawrenson, B. Lumb and R. Apps)

The cerebellum is typically recognized for its role in movement coordination and motor learning, but increasing evidence suggests it may also be involved in higher-order functions, including emotional behavior [4, 242, 243]. As described in the previous section, anatomical pathway tracing studies in animals have found that the cerebellum projects to an extensive list of brainstem and diencephalon nuclei, including a number of limbic structures [244–247]. In humans, structural and functional abnormalities can sometimes lead to impaired mood regulation and anxiety disorders (the cerebellar cognitive affect syndrome) [248–252]. In addition, neuroimaging studies have found changes in BOLD signal in the human cerebellum during fear learning paradigms [for review see 253]. In many cases, such changes are associated with the midline cerebellar vermis [249, 254, 255], and experimental studies in animals have found that lesions and other interventions of this cerebellar compartment have effects on defensive behaviors evoked by emotionally salient events [256–264].

The cerebellar vermis primarily consists of the “A” module. Individual modules are defined by their olivo-cortico-nuclear projections. In the case of the A module, the cortical parasagittal zonal component receives olivocerebellar (climbing fiber) input from the caudal medial accessory olive, and the Purkinje cells (PCs) located within this region of cortex have a corticonuclear output to the medial (fastigial) cerebellar nucleus [8, 45, 265]. This medial nucleus has widespread connections to midbrain and cerebral cortical regions including the amygdala, hypothalamus, periaqueductal gray (PAG), striatum, prefrontal cortex, parietal cortex, and hippocampus [218, 245–247]. Other cerebellar modules (notably the lateral vermal B and paravermal C3 modules) have been shown to be subdivided into smaller units, the cortical component of which are termed microzones [9, 27, 100, 101]. As discussed in previous sections, microzones and their micromodular connections are thought to represent the basic functional units of the cerebellum. Given that a finer grain olivocerebellar topography is present within the broader A module [266], this suggests a micromodular organization may also be present in this region of the cerebellum (see also section by Sugihara). Since emotional behaviors involve an integration of cognitive, somatomotor, and autonomic activity, this raises the possibility that different parts of the A module (possibly corresponding to micromodules) may be associated with different aspects of a coordinated emotional response.

To date, most studies have not attempted to explore cerebellar contributions to emotional behaviors at the modular (or micromodular) level of resolution. However, large cerebellar lesions involving vermal lobules III–VIII have shown various behavioral changes in relation to fearful or predator-prey interactions in rats. These include (i) fewer signs of fear when animals were placed in a brightly lit arena versus a dimly lit arena; (ii) decreases in freezing behavior and other signs of fear in the presence of a cat; (iii) faster recovery time than controls to the neophobic response to a novel taste test; and (v) attenuated spontaneous predation of mice [261, 264].

Some evidence for a lobular organization has also been found. For example, lesions of the anterior cerebellar vermis (lobules III–VI), but not the hemispheres resulted in impaired acquisition and retention of fear-conditioned bradycardia [256, 257]. Moreover, a subpopulation of PCs was found to respond to the conditioned stimulus, and in some cases this activity was correlated with the magnitude of the conditioned bradycardia response [267]. However, Supple et al. [267] did not investigate the possibility of correlated activity with other aspects of defensive behaviors, which might be expected if a finer grain localization of function was present.

In relation to human studies, a meta-analysis of fMRI mapping of the cerebellum supports a role for the human anterior lobe (vermal lobules IV and V) in fear learning and affective state [see review by 251]. And Sacchetti and colleagues found that reversible inactivation of a similar region in rats impairs the consolidation of fear memories [259, 263, 268]. By comparison, regions of the posterior lobe vermis appear to be involved in different aspects of behavior. In particular, it has long been known that vermal lobules VI and VII are important in the control of saccadic eye and head movements in a range of species including humans (the oculomotor vermis) [239, 269–273], while lesions of vermal lobule VIII in rats results in deficits in innate and conditioned fear induced freezing behavior but no detectable changes in general motor activity [258, 274]. By contrast, vermal lobules IX and X are related to autonomic functions including regulation of blood pressure, heart rate, respiration, and the baroreceptor reflex [275, 276].

**Fig. 8:**
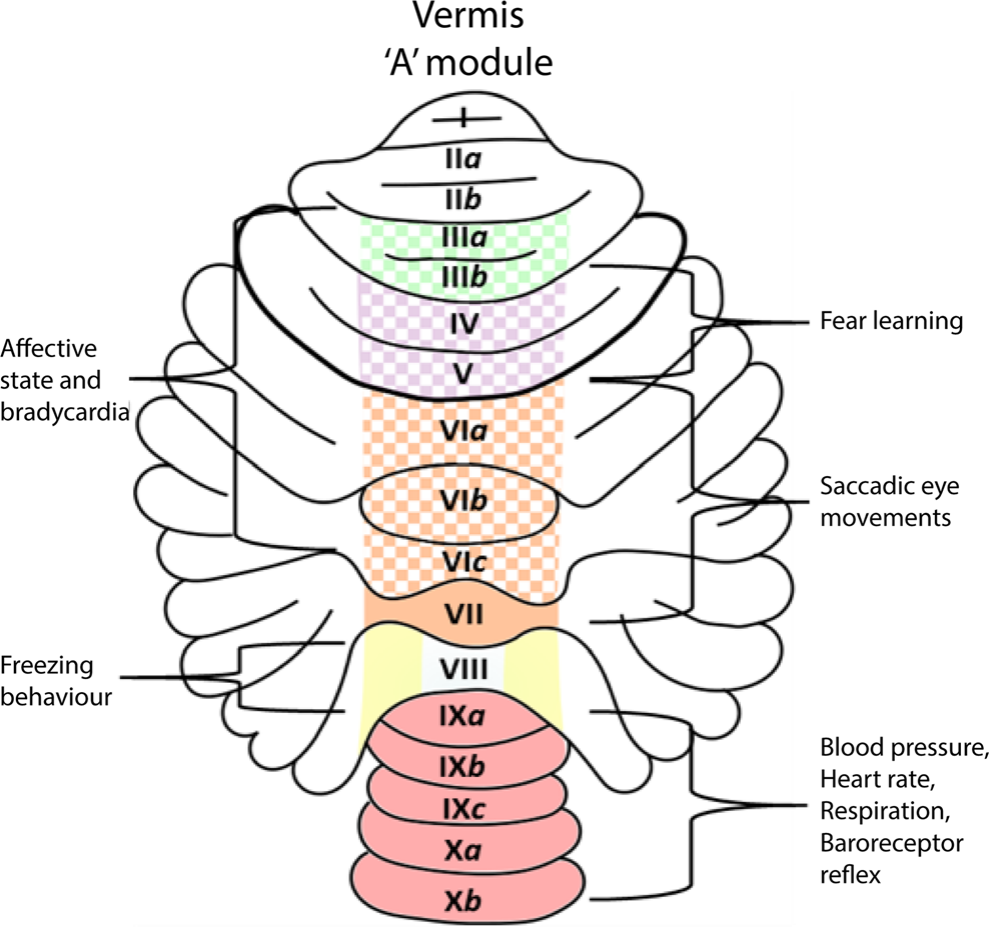
The “A” module of the cerebellar vermis can be separated into several different rostrocaudally arranged regions, in some cases corresponding to specific lobules, that are associated with a variety of cognitive, motor and autonomic functions relating to defensive behaviors

Thus, different cerebellar vermal lobules (that may relate to different components of the A module) appear to be associated with different aspects of an integrated array of behaviors. From rostral to caudal: lobules IV–VI with fear memory and affective state; lobule VI–VII with orientation of gaze; lobule VIII with fear-induced freezing behavior; and lobule IX and X with cardiorespiratory control (Fig. 8). Different parts of the A module, possibly relating to individual micromodules, could therefore regulate and integrate the cognitive, motor, and autonomic aspects of fear-related behavior.

## General Conclusions

The present collection of views on the anatomical and functional organization of the cerebellum has resulted in the realization that as a first requirement for a general consensus would be to agree to a general terminology. In this review, the authors agreed to use the following definitions of terms.

- Module: interconnected longitudinal zone of PCs (a sagittal zone), (large part of) cerebellar nucleus, and (large part) of olivary subnucleus generally referred by a letter and number of the participating sagittal zone (see next item)
- Sagittal zone: (para-)sagittal band of Purkinje cells with similar anatomical connections to a particular cerebellar or vestibular (sub-) nucleus and identified by a capital letter and number according to Voogd
- Microzone: PC zone with similar olivary receptive fields
- Micromodule/microcomplex: a microzone with its (potentially) interconnected small parts of a cerebellar nucleus and olivary subnucleus
- Quantum/super PC: smallest interconnected olivo-cortico-nuclear entity (with a similar function?)
- Stripe: chemically identified banding pattern of cerebellar cortex (usually identifying zebrin II/aldolase c bands)
- Strip/band/patch: array of PCs or mossy fibers within a zone or stripe or without a reference
- Transverse zone: one of four antero-posterior zebrin domains as defined by Hawkes

There is broad consensus that this battery of terms reflects different ways of describing and identifying the same complex modular architecture. First, there is the olivocerebellar projection pattern—separated into the cortical climbing fibers and their nuclear collaterals—which matches the corticonuclear projections of the targeted PCs. Matching nucleo-olivary projections close the modular loop, while the bulbar projections of the other nuclear neurons form the executive branch of such a module (Sugihara, Voogd: Fig. 1). Beyond this, cerebellar architecture at higher resolution is the topic of intense debate.

Although it is evident that there is generally a good correspondence between the chemical signature of Purkinje cells and their anatomical connections, the level of chemical complexity surpasses that of our current anatomical knowledge. To what extent are modules subdivided into smaller anatomical entities and can these really be seen as micromodules (Apps, Wylie)? What is the smallest operational unit in which the modular connectivity pattern can be recognized (Hawkes, Jörntell) and could this be different for different cerebellar modules (Hawkes, Apps)? Would such a cerebellar quantum or super PC require an anatomically fully closed integrated circuit between the incorporated inferior olivary cells, Purkinje cells, and nuclear cells? Another aspect that is still not fully understood is the anatomical incorporation of the nucleo-cortical connections into the modular circuitry [224, 277–279].

Questions concerning the anatomical equivalency of the relation between olivocorticonuclear connections and chemical identity of Purkinje cells also relate to the much more difficult question concerning the functional interpretation of cerebellar modules, as it is far from settled to what extent the olivocorticonuclear circuitry (as modules or micromodules) represent functional entities. This will necessitate a better understanding of how the organization of mossy fibers fits in the modular scheme (Hawkes, Sillitoe, Isope)? Questions of how the distributed mossy fiber-parallel fiber system functionally and adaptively interacts with individual (micro-)modules are far from answered (Ebner, Isope)? Likewise, it remains to be determined to what extent information processing in modules with different chemical signatures fundamentally differs (Schonewille, Ebner) and how this subsequently may be used in the same functional setting (Wylie, Ruigrok)? What, really, is the function of individual (micro) modules? Do modules cooperate both within and outside of the cerebellum and in what way? Are the different modules with the same basic connectivity (e.g., C1 and C3) signs of redundancy (Hawkes, Isope)? To what extent do cerebellar modules serve multiple but integrated functions (Apps)?

Although, these and other questions cannot yet be readily answered, hypotheses have been formulated and a host of new and innovative techniques are at hand to begin to explore them all. For now, it should be clear that the cerebellum cannot be seen as a single operational machine, but that its basic modular organization has the potential to serve a great many functions in both individual and integrated ways.
